# Hydrogen sulfide inhibits skeletal muscle ageing by up‐regulating autophagy through promoting deubiquitination of adenosine 5’‐monophosphate (AMP)‐activated protein kinase α1 via ubiquitin specific peptidase 5

**DOI:** 10.1002/jcsm.13560

**Published:** 2024-08-27

**Authors:** Jia‐He Yang, Jun Gao, Ya‐Qi E, Li‐Jie Jiao, Ren Wu, Qiu‐Yi Yan, Zi‐Yi Wei, Guo‐Liang Yan, Jin‐Long Liang, Hong‐Zhu Li

**Affiliations:** ^1^ Institute of Cardiovascular Diseases, Xiamen Cardiovascular Hospital of Xiamen University, School of Medicine Xiamen University Xiamen Fujian China; ^2^ Department of Pathophysiology, School of Medicine Xiamen University Xiamen Fujian China; ^3^ Department of Emergency Medicine Xiangan Hospital of Xiamen University Xiamen Fujian China; ^4^ Department of General Surgery Xiamen Fifth Hospital Xiamen Fujian China

**Keywords:** ageing, autophagy, deubiquitination, hydrogen sulfide, skeletal muscle

## Abstract

**Background:**

Hydrogen sulfide (H_2_S), the third gasotransmitter discovered, regulates a variety of physiological functions. Whether H_2_S alleviates skeletal muscle ageing by regulating autophagy has not been reported.

**Methods:**

Mice were administered 150 mg/kg/day of d‐galactose (d‐gal), and C2C12 myotubes were cultured in 20 g/L d‐gal to induce ageing. Sodium hydrosulfide (NaHS) was employed as an exogenous donor in the treatment group. The intracellular concentration of H_2_S was quantified by the 7‐azido‐4‐methylcoumarin fluorescence probe. The proteins involved in the ubiquitin‐mediated degradation of AMPKα1 were detected by liquid chromatography tandem mass spectrometry (LC–MS/MS) and co‐immunoprecipitation (Co‐IP). S‐sulfhydration of USP5 was tested by a biotin‐switch assay. Associated proteins were analysed by western blot.

**Results:**

NaHS was found to effectively restore the H_2_S content in both ageing gastrocnemius (+91.89%, *P* < 0.001) and C2C12 myotubes (+27.55%, *P* < 0.001). In comparison to the D‐gal group, NaHS was observed to increase the mean cross‐sectional area of muscle fibres (+44.91%, *P* < 0.001), to decrease the collagen volume fraction of gastrocnemius (−81.32%, *P* = 0.001) and to reduce the β‐galactosidase‐positive area of C2C12 myotubes (−28.74%, *P* < 0.001). NaHS was also found to reverse the expression of muscle atrophy F box protein (MAFbx), muscle‐specific RING finger protein 1 (MuRF1), Cyclin D1 and p21 in the ageing gastrocnemius tissue (MAFbx: −31.73%, *P* = 0.008; MuRF1: −32.37%, *P* = 0.003; Cyclin D1: +45.34%, *P* = 0.010; p21: −25.53%, *P* = 0.022) and C2C12 myotubes (MAFbx: −16.38%, *P* < 0.001; MuRF1: −16.45%, *P* = 0.003; Cyclin D1: +40.23%, *P* < 0.001; p21: −35.85%, *P* = 0.026). The AMPKα1–ULK1 pathway was activated and autophagy was up‐regulated in NaHS‐treated gastrocnemius tissue (p‐AMPKα1: +61.61%, *P* = 0.018; AMPKα1: +30.64%, *P* = 0.010; p‐ULK1/ULK1: +85.87%, *P* = 0.005; p62: −29.07%, *P* < 0.001; Beclin1: +24.75%, *P* = 0.007; light chain 3 II/I [LC3 II/I]: +55.78%, *P* = 0.004) and C2C12 myotubes (p‐AMPKα1: +77.49%, *P* = 0.018; AMPKα1: +26.18%, *P* = 0.022; p‐ULK1/ULK1: +38.34%, *P* = 0.012; p62: −9.02%, *P* = 0.014; Beclin1: +13.36%, *P* < 0.001; LC3 II/I: +79.38%, *P* = 0.017; autophagy flux: +24.88%, *P* = 0.034) compared with the d‐gal group. The effects of NaHS on autophagy were comparable to those of acadesine and LYN‐1604, and chloroquine could reverse its effects on ageing. LC–MS/MS and Co‐IP experiments demonstrated that USP5 is a deubiquitinating enzyme of AMPKα1. Following the knockdown of USP5, the activation of AMPKα1 was decreased (p‐AMPKα1: −42.10%, *P* < 0.001; AMPKα1: −43.93%, *P* < 0.001), autophagy was inhibited (p‐ULK1/ULK1: −27.51, *P* = 0.001; p62: +36.00, *P* < 0.001; Beclin1: −22.15%, *P* < 0.001) and NaHS lost its ability to up‐regulate autophagy. NaHS was observed to restore the expression (gastrocnemius: +62.17%, *P* < 0.001; C2C12 myotubes: +37.51%, *P* = 0.003) and S‐sulfhydration (+53.07%, *P* = 0.009) of USP5 and reduce the ubiquitination of AMPKα1.

**Conclusions:**

H_2_S promotes the deubiquitination of AMPKα1 by increasing the expression and S‐sulfhydration of USP5, thereby up‐regulating autophagy and alleviating skeletal muscle ageing.

## Introduction

The process of ageing is characterized by a gradual reduction in the body's capacity to adapt to environmental changes, which can ultimately lead to disability or even death. Skeletal muscle constitutes approximately 30–40% of body weight,^1^ and the majority of activities necessitate muscular contraction for completion. As individuals age, there is a concomitant decline in the number of muscle fibres and the cross‐sectional area of muscle.^2^ The decline in mass and function of skeletal muscle in older adults often results in falls, disability and even death.^3^ It is of paramount importance to alleviate the ageing process of skeletal muscle in order to extend the lifespan of elderly individuals.

Hydrogen sulfide (H_2_S) is a gasotransmitter that is produced endogenously in mammals, primarily through enzymatic pathways.^4^ H_2_S directly reacts with oxygen, hydrogen peroxide and peroxynitrite, thereby reducing cellular oxidative damage. Additionally, it can modify proteins post‐translationally through S‐sulfhydration, which affects their functionality.^5^ Studies have demonstrated that human skeletal muscle expresses a considerable number of H_2_S‐producing enzymes, including cystathionine‐γ‐lyase (CSE), cystathionine‐β‐synthetase (CBS) and 3‐mercaptopyruvate sulfurtransferase (3‐MST).^6^ H_2_S has been shown to effectively alleviate muscle atrophy caused by diabetes and obesity.^7^ The precise mechanism by which this occurs is not yet fully understood. However, scientists have postulated that it may be related to H_2_S antioxidant stress, the regulation of mitochondrial energy metabolism, the reduction of apoptosis and the up‐regulation of autophagy.^6^

Autophagy is a cellular process that degrades and recycles a diverse array of cellular contents, including proteins, organelles and even pathogens.^8^ Dysfunctional autophagy results in protein imbalance and energy metabolism disorders, thereby accelerating cellular ageing.^9^ Studies have demonstrated that restoration of the autophagy‐related protein expression, including adenosine 5′‐monophosphate (AMP)‐activated protein kinase (AMPK), autophagy protein 18 (ATG18) and ATG8a, can prolong the lifespan of *Caenorhabditis elegans* and drosophila.^10^, ^11^ Previous studies conducted by our research group have demonstrated that H_2_S inhibits ageing in the heart^12^ and kidneys^13^ by up‐regulating autophagy. These studies indicate that enhancing autophagy may be a pivotal mechanism by which H_2_S delays skeletal muscle ageing.

AMPK, composed of α, β and γ subunits, is encoded by the α1, α2, β1, β2, γ1, γ2 and γ3 genes, which plays an important role in the initiation of autophagy. Activation of AMPKα1 results in the phosphorylation of uncoordinated‐51‐like kinase 1 (ULK1), which then binds to ATG13, ATG101 and FAK‐family interacting protein 200 to initiate autophagy.^14^ In addition, AMPK can also increase S90 and S93 phosphorylation of Beclin1, thus promoting autophagosome nucleation.^15^ The majority of current studies on AMPK focus on its activation by phosphorylation and subsequent degradation by ubiquitination. Moreover, it has been demonstrated that AMPK can be regulated by deubiquitination.^16^

Ubiquitin‐specific proteases (USPs) constitute a subclass of deubiquitination enzymes (DUBs), with ubiquitin‐specific peptidase 5 (USP5) representing a pivotal member of USPs.^17^ The current literature on USP5 is largely focused on studies of cancer and inflammation. Studies have shown that USP5 can deubiquitinate Forkhead Box M 1 (FoxM1) and NLR family pyrin domain containing 3 (NLRP3), which promotes the growth of pancreatic ductal adenocarcinoma and non‐small cell lung cancers and reduce inflammation.^18^, ^19^ In a separate study, Li et al.^20^ found that USP5 can also deubiquitinate Beclin1, thereby maintaining autophagy levels. However, the question of whether USP5 regulates AMPKα1 through deubiquitination remains unanswered. The objective of this study is to investigate whether H_2_S can enhance the expression and S‐sulfhydration of USP5, thereby facilitating the deubiquitination of AMPKα1. Which, in turn, would result in the up‐regulation of autophagy, which would contribute to the alleviation of skeletal muscle ageing.

## Materials and methods

### Reagents

The detailed account of the reagents utilized in this study is shown in *Method S1*.

### Animal model and treatment protocols

All male C57BL/6J mice were obtained from the Experimental Animal Center of Xiamen University (SPF grade). A total of 60 mice, aged 8–9 weeks, were randomly divided into three groups: (1) control group: the mice were injected with an equivalent volume of normal saline for 10 consecutive weeks; (2) d‐galactose (d‐gal) group: the mice were subcutaneously injected with 150 mg/kg/day of d‐gal for 10 consecutive weeks; and (3) D‐gal + sodium hydrosulfide (NaHS) group: the mice were injected intraperitoneally with 100 μmol/kg/day NaHS 1 h after d‐gal injection for 10 consecutive weeks. Twenty mice aged 8–9 weeks and 40 mice aged 15 months were divided into three groups: (1) young group: mice aged 8–9 weeks received no treatment; (2) old group: the 15‐month‐old mice were fed for 3 months without any other treatment; and (3) old + NaHS group: the 15‐month‐old mice were administered 100 μmol/kg/day NaHS via intraperitoneal injection for a period of three consecutive months. All animal experiments were conducted in accordance with the Animal Management Regulations of the Ministry of Science and Technology of China and approved by the Experimental Animal Ethics Committee of Xiamen University.

### Cell culture and treatment

The mouse C2C12 myoblast line was obtained from Professor Lin Donghai's laboratory at Xiamen University and was purchased from the Chinese Academy of Sciences (Shanghai, China). The cells were cultured in Dulbecco's modified Eagle's medium (DMEM) containing 10% foetal bovine serum (FBS) and 1% antibiotics (100 U/mL of penicillin and 10 mg/mL of streptomycin). Subsequently, the cells were cultured in DMEM containing 2% horse serum for 7 days, during which time the medium was changed every 48 h to differentiate C2C12 myoblasts into C2C12 myotubes. C2C12 myotubes were subsequently divided into the following groups: control group, d‐gal group (20 g/L d‐gal), d‐gal + NaHS (20 g/L d‐gal, 150 μM NaHS), d‐gal + chloroquine (CQ) group (pretreated with 40 μM CQ for 4 h, 20 g/L d‐gal), d‐gal + NaHS + CQ group (pretreated with 40 μM CQ for 4 h, 20 g/L d‐gal, 150 μM NaHS), d‐gal + acadesine (AICAR, 20 g/L d‐gal, 1 mM AICAR was added 4 h before sample collection), d‐gal + LYN‐1604 (20 g/L d‐gal, 2 μM LYN‐1604 was added 4 h before sample collection) and d‐gal + de‐sulfhydration reagent dithiothreitol (DTT, 20 g/L d‐gal, 200 μM DTT was added 2 h before sample collection). All the cells were cultured in 2% FBS for 48 h.

### Western blot

The procedure is shown in *Method* *S2*.

### Co‐immunoprecipitation (Co‐IP)

The appropriate amount of lysis buffer (1% PMSF, 1% PIC) was added to the samples of C2C12 myotubes. The samples were disrupted and centrifuged, after which the supernatant was extracted. The homogenate was diluted to a concentration of 3 μg/μL in a protein system comprising 300 μL. Each sample was then treated with 3 μg anti‐IgG and 20 μL protein A/G magnetic beads to remove non‐specific binding proteins. Subsequently, anti‐AMPKα1 (3 μg), anti‐USP5 (3 μg) or anti‐IgG (3 μg) was added to the samples in accordance with the instructions, and the samples were rotated at 4°C overnight. A total of 30 μL of protein A/G magnetic beads were added to the samples and rotated at 4°C for 3 h. The protein bound to the magnetic beads was eluted using 1% sodium dodecyl sulfate (SDS) sample buffer, and the protein interaction was then examined by western blot.

### Immunofluorescence

The procedure is shown in *Method* *S3*.

### Hydrogen sulfide content determination

The detection of H_2_S content in gastrocnemius and C2C12 myotubes was achieved through the use of 7‐azido‐4‐methylcoumarin (C‐7Az). Following the removal of any residual tissue debris from the slices and cells via PBS‐based washing, the samples were incubated with 50 μM C‐7Az for 30 min. Subsequently, the samples were washed with PBS three times. The fluorescence intensity was then observed under a fluorescence microscope.

### Senescence‐associated β‐galactosidase (SA‐β‐gal) staining

The procedure is shown in *Method* *S4*.

### Haematoxylin and eosin (H&E) and Masson staining

The procedure is shown in *Method* *S5*.

### Biochemical assays

The procedure is shown in *Method* *S6*.

### Reactive oxygen species (ROS) content determination

The procedure is shown in *Method* *S7*.

### TUNEL and Hoechst 33342 staining

The procedure is shown in *Method* *S8*.

### Transmission electron microscopy

The procedure is shown in *Method* *S9*.

### Monodansylcadaverine (MDC) staining

The procedure is shown in *Method* *S10*.

### S‐sulfhydration assay of ubiquitin specific peptidase 5

The cells of different groups were lysed in 200 μL HEN lysis buffer (250 mM Hepes‐NaOH, 1 mM EDTA, 0.1 mM neocuproine, 150 μM deferoxamine, 1% NP 40). The samples were then centrifuged at 4°C and 12 000 rpm for 10 min, after which the supernatant was collected. Each group of samples was then added to 1 mL of blocking buffer (2.5% SDS and 20 mM methylethylthionate) and incubated at 50°C for 30 min. Each group of sealed liquid was reacted with 5 mL of acetone at a temperature of −20°C for a period of 60 min. Subsequently, the supernatant was removed by centrifugation at 3000 rpm for 5 min, and the precipitate was re‐suspended in the biotin‐HPDP‐HENS buffer liquid system for 3 h. Target proteins were precipitated with streptavidin magnetic beads and rotated at 4°C overnight. On the following day, the beads were washed three times with PBS with Tween 20 (PBST) buffer. The magnetic beads were then eluted with 20 mM DTT solution and sodium dodecyl sulfate polyacrylamide gel electrophoresis (SDS‐PAGE) sample buffer. The content of biotinylated USP5 in the eluted samples was then detected by western blot.

### Protein mass spectrometric analysis

The separation of proteins was achieved through the use of SDS‐PAGE, with the gels subsequently being cut. The gels were stained with Coomassie blue solution for 30 min and washed with decolorizing solution (40% acetonitrile, 50 mM ammonium bicarbonate) for 30–60 min. They were then rinsed with double‐distilled water for 2 h. Finally, the gels were cut into 1.5 mm × 1.5 mm pieces and subjected to in‐gel digestion and detection with liquid chromatography tandem mass spectrometry (LC–MS/MS). Protein identification was performed using Thermo Orbitrap Fusion Lumos and its built‐in database. The identification results are considered valid when a unique peptide of a protein is found and is not present in the native control group.

### siRNA transfection

The sequences of the siRNA are as follows: mice USP5 siRNA (#1 S: 5′‐GCCUCUAUAUCUGCAUGAATT‐3′ AS: 5′‐UUCAUGCAGAUAUAGAGGCTT‐3′, #2 S: 5′‐CCGGAAAGCUGUGUACUAUTT‐3′ AS: 5′‐AUAGUACACAGCUUUCCGGTT‐3′), mice negative control siRNA (S: 5′‐UUCUCCGAACGUGUCACGUTT‐3′ AS: 5′‐ACGUGACACGUUCGGAGAATT‐3′). The specific transfection method is shown in *Method* *S11*.

### Statistical analysis

All data were expressed as the mean ± standard error of the mean (SEM). In instances where the data exhibited variance homogeneity, an ordinary one‐way analysis of variance (ANOVA) was employed for analysis. Conversely, when the data did not meet the requisite variance homogeneity, a Kruskal–Wallis test was utilized. All images were analysed using ImageJ, and the results were subsequently analysed using GraphPad Prism 8 (San Diego, CA, USA). Tukey's post hoc test was employed to ascertain the statistical significance of the results, with *P* ≤ 0.05 deemed to be statistically significant. No electronic laboratory notebook was utilized.

## Results

### d‐Galactose induces ageing, and sodium hydrosulfide up‐regulates hydrogen sulfide levels in the skeletal muscle of mice and C2C12 myotubes

The expression of cyclin‐dependent kinase inhibitor 1A (p21) and Cyclin D1 was detected, which reflects the progression of the cell cycle.^21^ The results demonstrated that, in comparison to young mice, the expression of p21 was increased and the expression of Cyclin D1 was decreased in d‐gal‐induced ageing mice (*Figure* *S1* *a*). C2C12 myoblasts were observed to differentiate into C2C12 myotubes following a 7‐day incubation period in differential medium (*Figure* *S1* *b*). The expression of Cyclin D1 was reduced and the expression of p21 was increased following treatment with 20 g/L d‐gal for 48 h (*Figure* *S1* *c*). Consequently, a concentration of 20 g/L d‐gal was employed in subsequent experiments.

**Figure 1:**
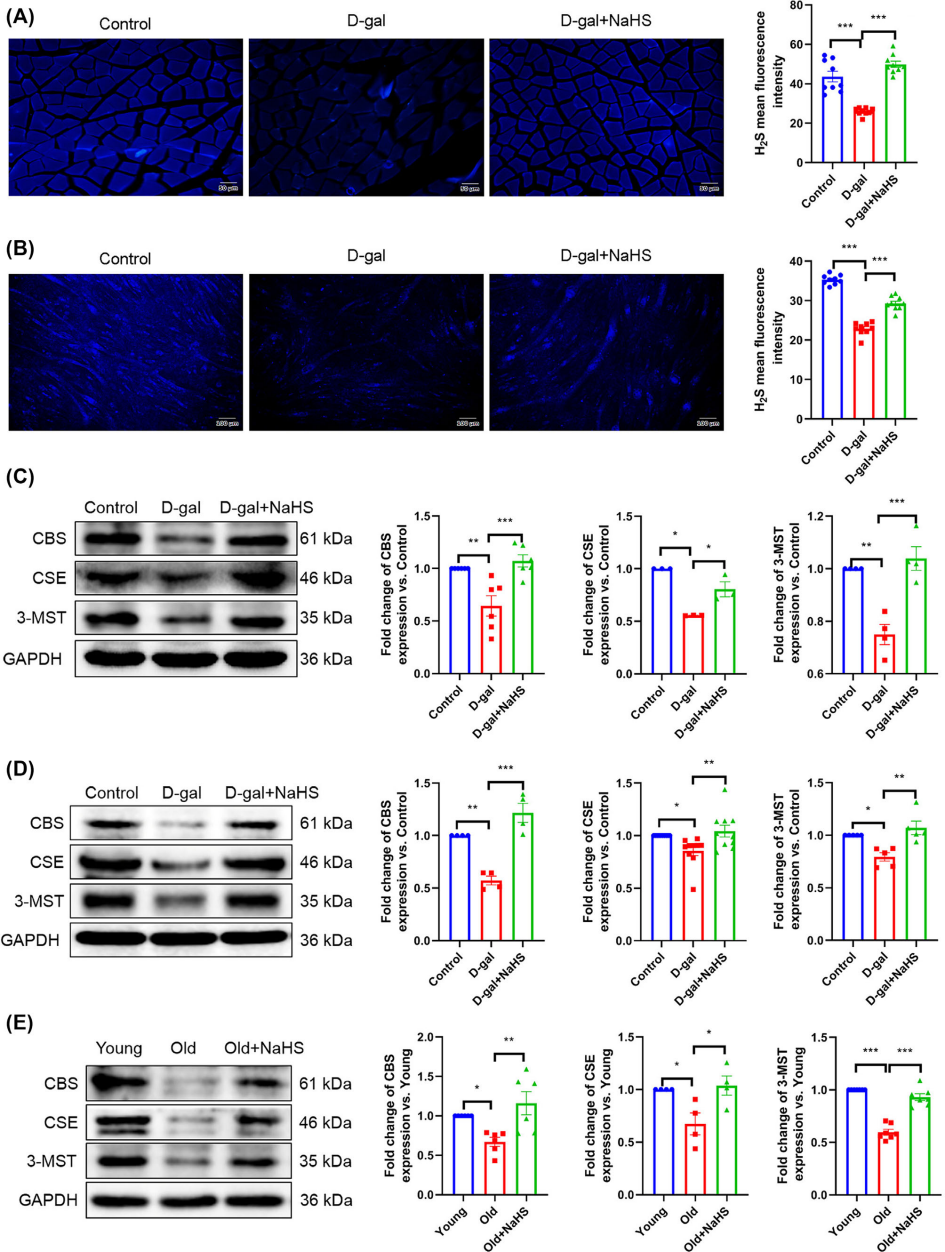
NaHS increases H_2_S content and the expression of H_2_S‐producing enzymes in ageing skeletal muscle tissue and C2C12 myotubes. (A, B) H_2_S (blue) levels in gastrocnemius tissue (A, scale bar, 50 μm; magnification, ×200, *n* = 9) and C2C12 myotubes (B, scale bar, 100 μm; magnification, ×100, *n* = 9) as assessed by the fluorescence probe C‐7Az. (C) The expression of CBS, CSE and 3‐MST in d‐gal‐induced gastrocnemius tissue (*n* ≥ 3) was detected by western blot. (D) The expression of CBS, CSE and 3‐MST in C2C12 myotubes (*n* ≥ 4) was detected by western blot. (E) The expression of CBS, CSE and 3‐MST in natural ageing gastrocnemius tissue (*n* ≥ 4) was detected by western blot. The results were expressed as the mean ± SEM. Significant differences are indicated as *P < 0.05, **P < 0.01 and ***P < 0.001. 3‐MST, 3‐mercaptopyruvate sulfurtransferase; CBS, cystathionine‐β‐synthetase; CSE, cystathionine‐γ‐lyase; d‐gal, d‐galactose; H_2_S, hydrogen sulfide; NaHS, sodium hydrosulfide; old, 18‐month‐old mice; young, 8‐ to 9‐week‐old mice.

Furthermore, our results indicate that the H_2_S content and the expression of CBS, CSE and 3‐MST were significantly decreased in the d‐gal group gastrocnemius (*Figure* *1* *A,C*) and C2C12 myotubes (*Figure* *1* *B,D*) in comparison to the control group. In contrast to the d‐gal group, NaHS increased the H_2_S content and the expression of CBS, CSE and 3‐MST in gastrocnemius (*Figure* *1* *A,C*) and C2C12 myotubes (*Figure* *1* *B,D*). Additionally, these results were verified in natural ageing mice (*Figure* *1* *E*). These findings indicate that skeletal muscle ageing is inextricably linked to a reduction in H_2_S production. NaHS has been demonstrated to up‐regulate H_2_S‐producing enzyme expression and H_2_S content.

### Hydrogen sulfide alleviates skeletal muscle ageing

**Figure 2:**
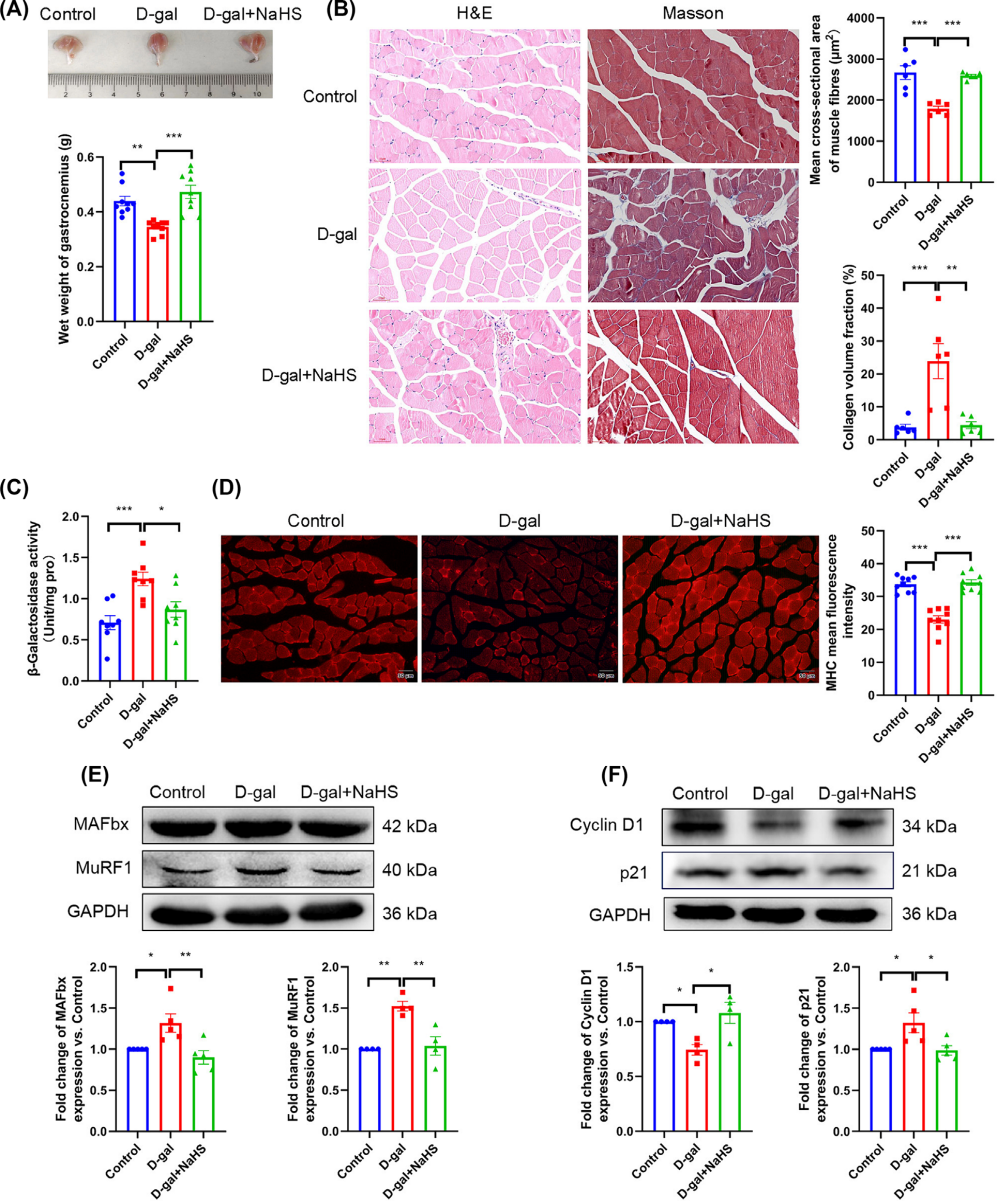
H_2_S alleviates skeletal muscle ageing. (A) The photograph and wet weight of isolated gastrocnemius from different groups (*n* = 9). (B) Morphology changes of gastrocnemius observed by H&E and Masson staining (scale bar, 50 μm; magnification, ×200, *n* = 6), the mean cross‐sectional area of muscle fibres and collagen volume fraction were detected by ImageJ. (C) SA‐β‐gal activity of gastrocnemius tissue was measured by biochemical assay kits (*n* = 9). (D) The positioning of MHC (red) in gastrocnemius tissue was measured by immunofluorescence (scale bar, 50 μm; magnification, ×200, *n* = 9). (E) Expression of MAFbx (*n* = 5) and MuRF1 (*n* = 4) in gastrocnemius tissue. (F) Expression of Cyclin D1 (*n* = 4) and p21 (*n* = 5) in gastrocnemius tissue. The results were expressed as the mean ± SEM. Significant differences are indicated as *P < 0.05, **P < 0.01 and ***P < 0.001. H&E, haematoxylin and eosin; MAFbx, muscle atrophy F box protein; MHC, myosin heavy chain; MuRF1, muscle‐specific RING finger protein 1; p21, cyclin‐dependent kinase inhibitor 1A; SA‐β‐gal, senescence β‐galactosidase.

To further substantiate the relationship between H_2_S and skeletal muscle ageing, we conducted the following experiments: The gastrocnemius of ageing mice exhibited clear signs of muscle atrophy, and NaHS was found to significantly inhibit muscle atrophy, as evidenced by the photographic images and wet weight of the gastrocnemius samples (*Figure* *2* *A*). The results of H&E staining demonstrated that skeletal muscle from ageing mice exhibited myolysis and a reduction in the mean cross‐sectional area of muscle fibres. The results of Masson staining demonstrated a significant increase in the collagen volume fraction of ageing skeletal muscle. The administration of NaHS was clearly effective in mitigating the aforementioned morphological changes associated with skeletal muscle ageing (*Figure* *2* *B*).

**Figure 3:**
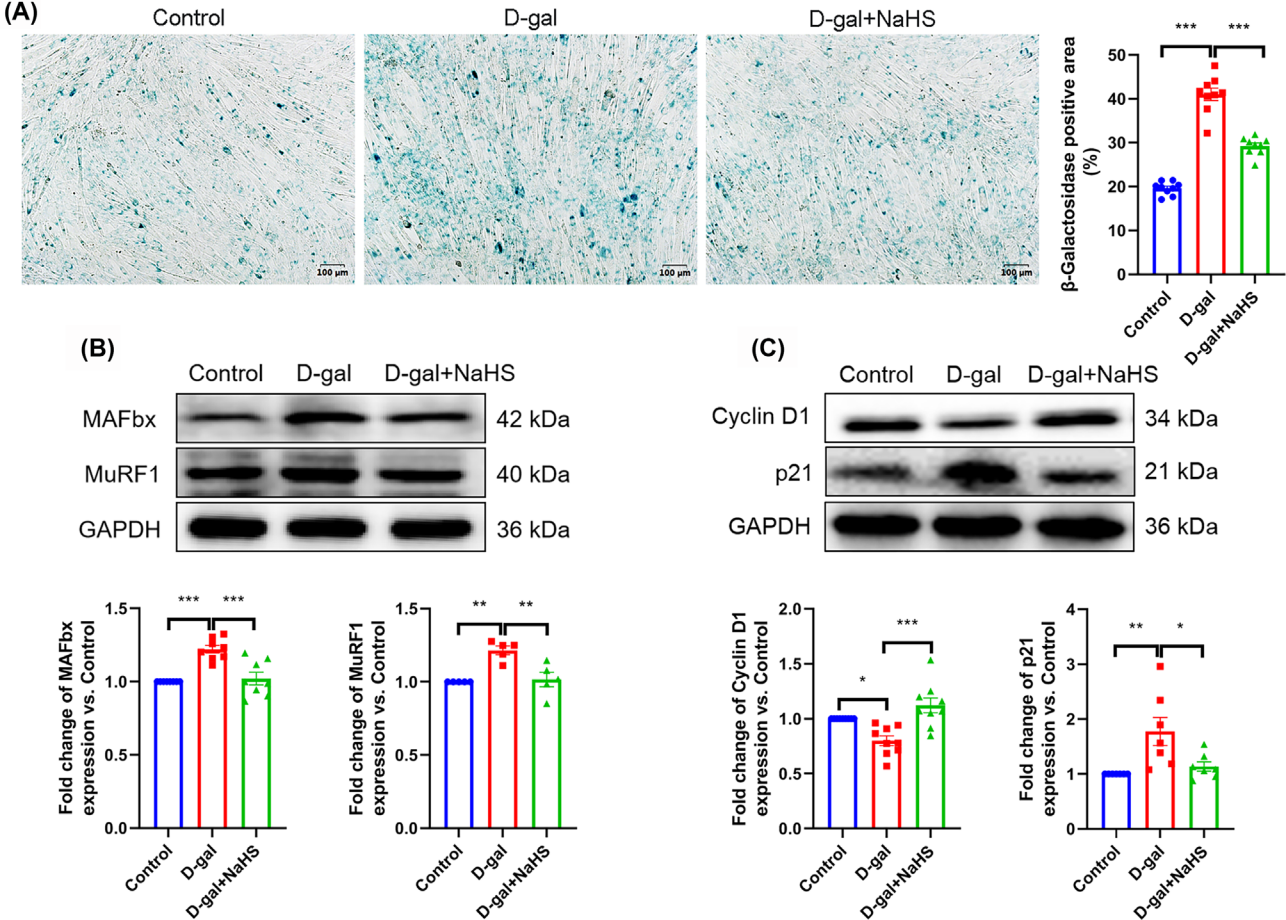
H_2_S alleviates C2C12 myotubes ageing. (A) Representative pictures of SA‐β‐gal staining in C2C12 myotubes; β‐galactosidase‐positive areas are blue (scale bar, 100 μm; magnification, ×100, *n* = 9). (B) The expression of MAFbx (*n* = 8) and MuRF1 (*n* = 5) in C2C12 myotubes. (C) The expression of Cyclin D1 (*n* = 9) and p21 (*n* = 7) in C2C12 myotubes. The results were expressed as the mean ± SEM. Significant differences are indicated as *P < 0.05, **P < 0.01 and ***P < 0.001.

Muscle‐specific RING finger protein 1 (MuRF1) and muscle atrophy F box protein (MAFbx) are two muscle‐specific E3 ubiquitin ligases.^22^ In the animal experiments, we found that the expression of Cyclin D1 was decreased, while the expression of MuRF1, MAFbx and p21 and the activity of SA‐β‐gal were enhanced in the d‐gal group (vs. the control group). In comparison to the d‐gal group, the NaHS treatment demonstrated a notable reversal of the observed alterations in the aforementioned indicators (*Figure* *2* *C–F*). All the above results were verified in cell experiments and natural ageing mice (*Figure* *3* and *S2a–c*). These data demonstrate that H_2_S significantly inhibits skeletal muscle ageing and atrophy in mice.

### Hydrogen sulfide relieves skeletal muscle ageing by inhibiting oxidative stress and apoptosis

Furthermore, ageing is associated with increased levels of oxidative stress and apoptosis.^23^ The levels of oxidative stress and apoptosis in gastrocnemius tissue and cells were examined, and it was found that the ROS and MDA contents were increased, while the SOD and CAT activities were reduced in the d‐gal group compared with the control group. In comparison to the d‐gal group, the d‐gal + NaHS group exhibited a notable reduction in ROS and MDA content and an enhancement in SOD and CAT activity (*Figure* *S3*). Furthermore, the number of apoptotic cells and the expression of Cleaved‐caspase9 and Cleaved‐caspase3 were higher, while the expression of B‐cell leukaemia/lymphoma 2 (Bcl2) was lower in the d‐gal group than in the control group. The effect of D‐gal on the aforementioned indicators was negated by NaHS (*Figure* *S4*). The experimental results suggest that H_2_S delays skeletal muscle ageing and atrophy by inhibiting oxidative stress and apoptosis in mice.

### Hydrogen sulfide inhibits skeletal muscle ageing by up‐regulating autophagy

**Figure 4:**
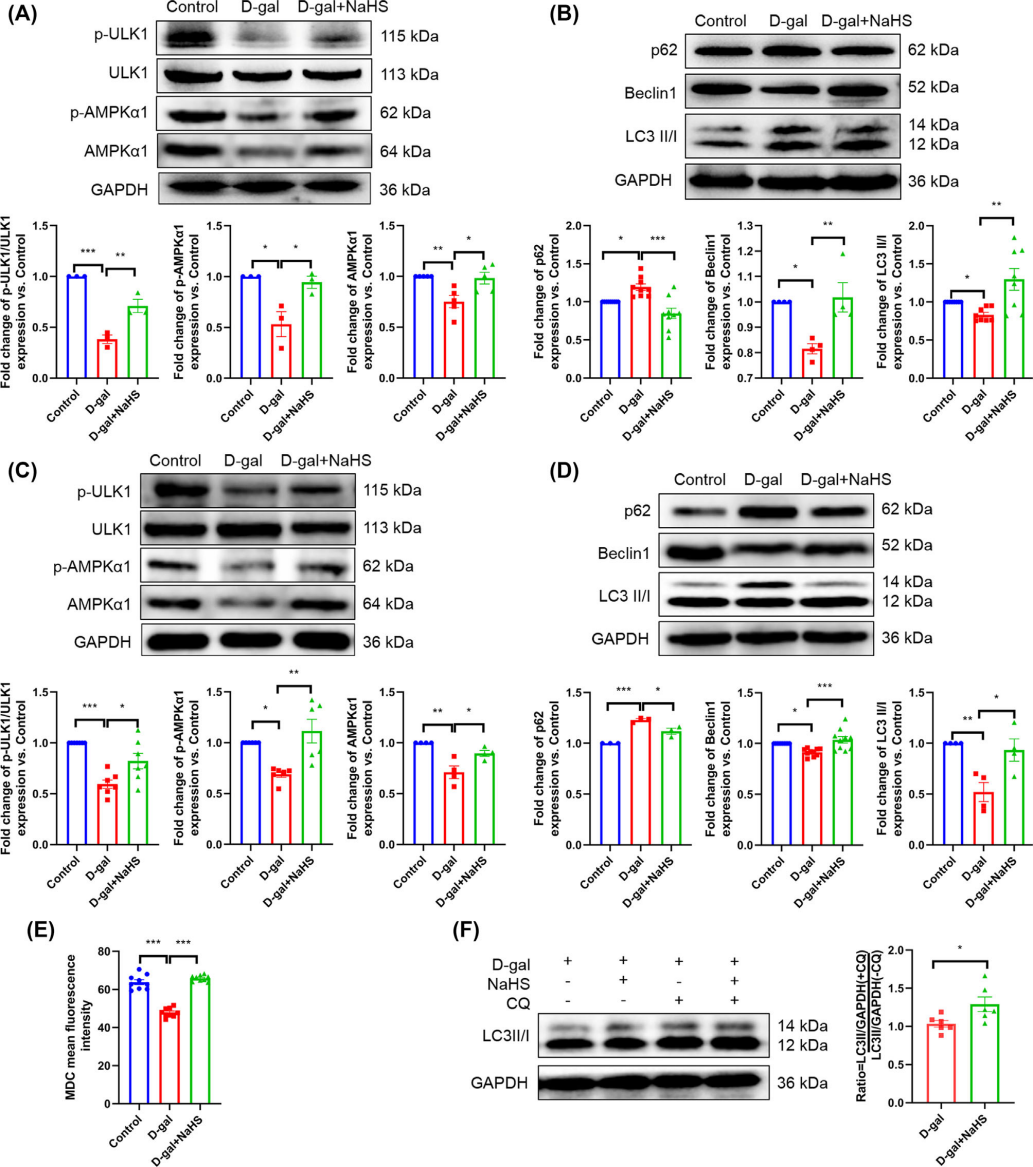
H_2_S up‐regulates autophagy in ageing skeletal muscle and C2C12 myotubes. (A, C) The level of p‐ULK1/ULK1, p‐AMPKα1 and AMPKα1 in gastrocnemius tissue (A, *n* ≥ 3) and C2C12 myotubes (C, *n* ≥ 4). (B, D) The expression of p62, Beclin1 and LC3 II/I in gastrocnemius tissue (B, *n* ≥ 4) and C2C12 myotubes (D, *n* ≥ 3). (E) Mean fluorescence intensity of MDC staining in C2C12 myotubes; representative pictures are provided in the supporting information (*n* = 9). (F) The level of autophagic flux in C2C12 myotubes (*n* = 6); samples were treated with or without the autophagy inhibitor chloroquine (CQ, 40 μM) for 4 h. The results were expressed as the mean ± SEM. Significant differences are indicated as *P < 0.05, **P < 0.01 and ***P < 0.001. AMPKα1, adenosine 5′‐monophosphate (AMP)‐activated protein kinase α1; CQ, chloroquine; LC3, microtubule‐associated protein 1 light chain 3; MDC, monodansylcadaverine; SQSTM1/p62, sequestosome 1; ULK1, uncoordinated‐51‐like kinase 1.

The relevant indicators of autophagy were examined in gastrocnemius tissue and C2C12 myotubes. The number of autophagosomes was found to be decreased, as was the expression of Beclin1 and the level of microtubule‐associated protein 1 light chain 3 II/I (LC3 II/I). In contrast, the expression of sequestosome 1 (SQSTM1/p62) was increased in gastrocnemius tissue from the d‐gal group. However, NaHS effectively reversed the changes in relevant indicators of autophagy in the ageing gastrocnemius tissue (*Figure* *4* *B* and *S5a*). We obtained consistent results at the cellular level (*Figure* *4* *D,E* and *S5b*). To assess the autophagic flux in myotubes, we employed the tool drug CQ. The results demonstrated that the autophagic flux in the d‐gal + NaHS group was higher than that in the d‐gal group (*Figure* *4* *F*). Subsequently, these results were verified in natural ageing mice (*Figure* *S2d*). These findings suggest a correlation between skeletal muscle ageing and the decline in autophagy and that H_2_S may inhibit skeletal muscle ageing by enhancing autophagy.

To further confirm the relationship between H_2_S resistance to muscle ageing and increased levels of autophagy, we pretreated C2C12 myotubes with 40 μM CQ for 4 h. The data showed that the number of autophagosomes and the expression of p62 were significantly increased in the D‐gal + NaHS + CQ group (vs. the d‐gal + NaHS group) (*Figure* *S6a,b*). Furthermore, we observed that CQ cancelled the impact of NaHS on skeletal muscle ageing‐related indicators, including the activity of SA‐β‐gal and the expression of MuRF1, MAFbx, Cyclin D1 and p21 (*Figure* *S6c–e*). All the above results indicate that H_2_S is unable to resist skeletal muscle ageing in the context of autophagy blockade. In other words, H_2_S inhibits skeletal muscle ageing by up‐regulating autophagy.

### Activation of the adenosine 5′‐monophosphate (AMP)‐activated protein kinase α1‐uncoordinated‐51‐like kinase 1 pathway by hydrogen sulfide up‐regulates autophagy

**Figure 5:**
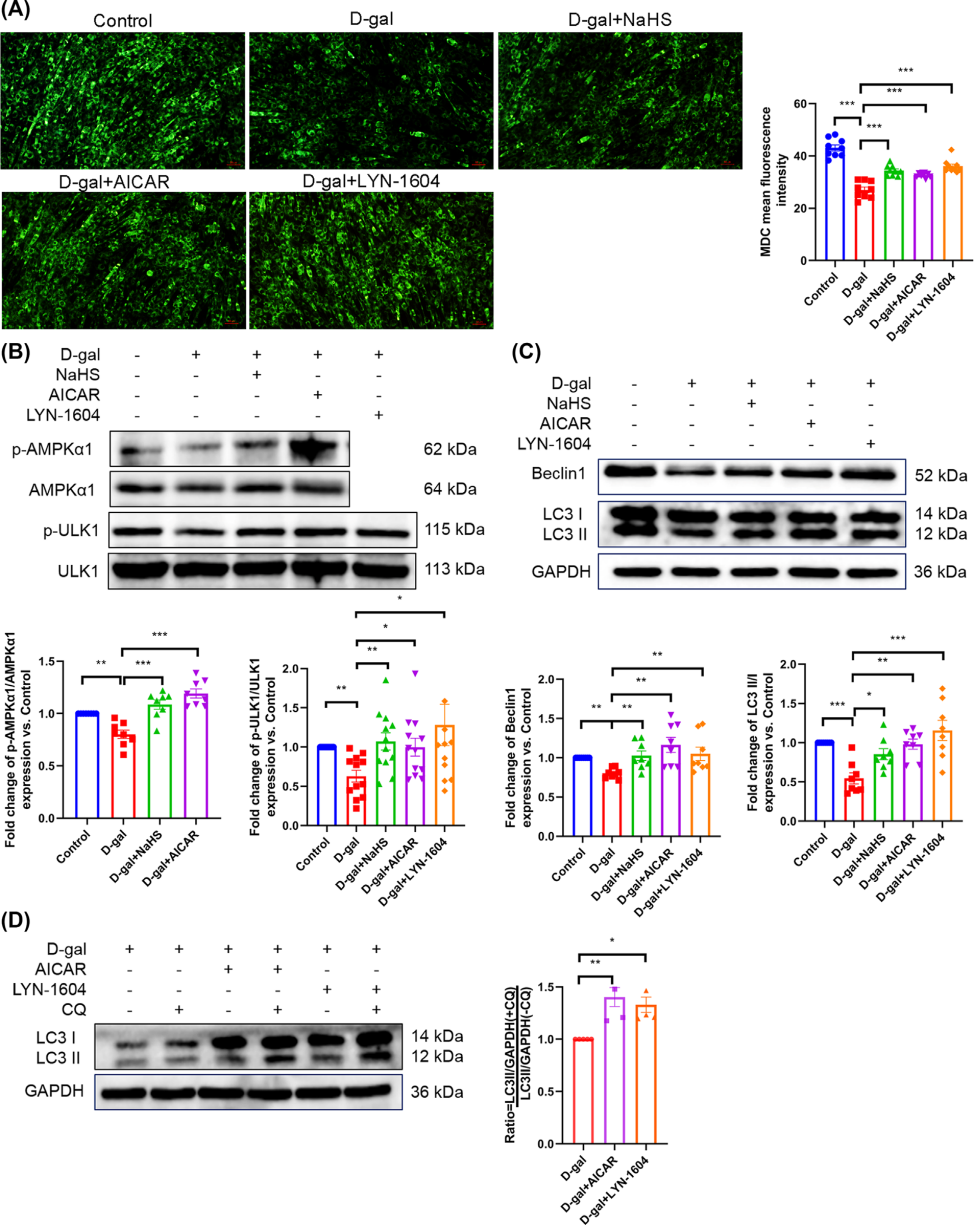
Activation of the AMPKα1–ULK1 pathway up‐regulates autophagy. (A) Autophagosomes (green) of C2C12 myotubes were detected by MDC staining (scale bar, 50 μm; magnification, ×100, *n* = 9). (B) The phosphorylation of ULK1 (*n* = 12) and AMPKα1 (*n* = 8) in C2C12 myotubes. (C) The expression of Beclin1 (*n* = 8) and LC3 II/I (*n* = 8) in C2C12 myotubes. (D) The level of autophagic flux in C2C12 myotubes (*n* = 4). Samples were treated with or without the autophagy inhibitor CQ (40 μM) for 4 h. The results were expressed as the mean ± SEM. Significant differences are indicated as *P < 0.05, **P < 0.01 and ***P < 0.001. AICAR, acadesine.

Phosphorylation of AMPK and ULK1 represents a pivotal step in the initiation of autophagy. The results demonstrated that the contents of p‐AMPKα1 and AMPKα1, as well as the levels of p‐ULK1/ULK1, were reduced in ageing gastrocnemius tissue (*Figure* *4* *A*) and C2C12 myotubes (*Figure* *4* *C*). Conversely, H_2_S was found to effectively increase the contents of p‐AMPKα1 and AMPKα1, as well as the level of p‐ULK1/ULK1 (*Figure* *4* *A,C*). The results of our experiments suggest that H_2_S up‐regulates autophagy by activating the AMPKα1–ULK1 pathway. To further substantiate this hypothesis, we employed AICAR (an AMPK agonist) and LYN‐1604 (a ULK1 agonist) as intervention factors. We found that, compared with the d‐gal group, the number of autophagosomes (*Figure* *5* *A*), the phosphorylation of ULK1, the expression of Beclin1 and LC3 II/I and the autophagic flux level were markedly increased in the D‐gal + AICAR and d‐gal + LYN‐1604 groups. Meanwhile, AICAR was observed to enhance the phosphorylation of AMPKα1. The effect of NaHS on the above indicators was similar to that of AICAR and LYN‐1604 (*Figure* *5* *B–D*). In addition, the experimental results demonstrated that NaHS, AICAR and LYN‐1604 exhibited a significant reduction in SA‐β‐gal activity when compared with d‐gal (*Figure* *S7*). These findings indicate that H_2_S activates the AMPKα1–ULK1 pathway to initiate autophagy, thereby alleviating skeletal muscle ageing.

### Ubiquitin specific peptidase 5 deubiquitinates adenosine 5′‐monophosphate (AMP)‐activated protein kinase α1 and then increases autophagy

**Figure 6:**
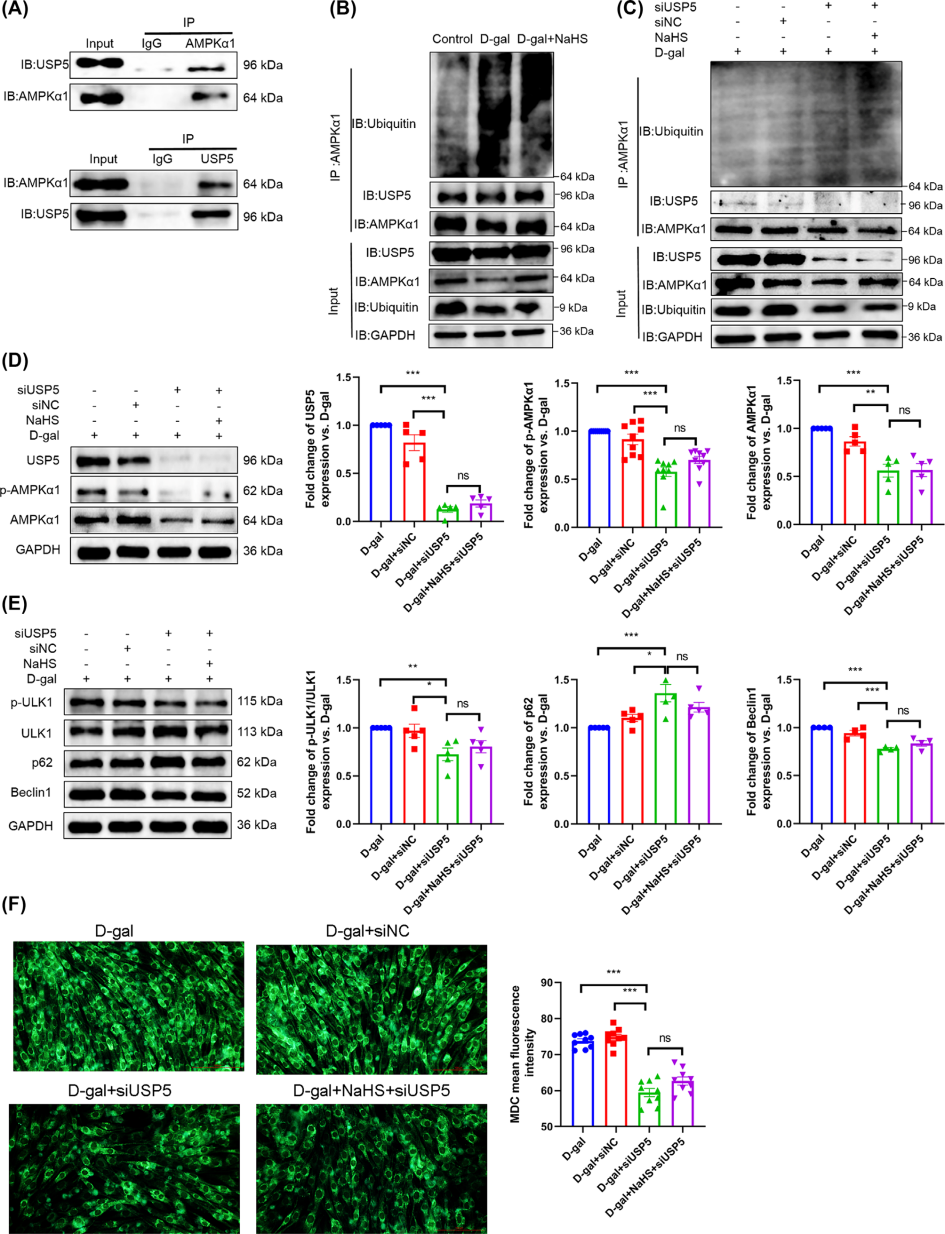
USP5 deubiquitinates AMPKα1 and then increases autophagy. (A) Interaction between USP5 and AMPKα1 was detected by Co‐IP. (B) Ubiquitination level of AMPKα1 and its interaction with USP5 in different groups (*n* = 3). (C) The ubiquitination level of AMPKα1 and its interaction with USP5 after knockdown of USP5 in C2C12 myoblasts (*n* = 3). (D) The expression of USP5 (*n* = 5), p‐AMPKα1 (*n* = 9) and AMPKα1 (*n* = 5) in C2C12 myoblasts. (E) The level of p‐ULK1/ULK1 (*n* = 5), the expression of p62 (*n* = 5) and Beclin1 (*n* = 4) in C2C12 myoblasts. (F) Autophagosomes (green) of the C2C12 myoblasts were detected by MDC staining (scale bar, 100 μm; magnification, ×200, *n* = 9). The results were expressed as the mean ± SEM. Significant differences are indicated as *P < 0.05, **P < 0.01 and ***P < 0.001. USP5, ubiquitin specific peptidase 5.

To elucidate the mechanism by which H_2_S modulates the AMPKα1–ULK1 pathway, we employed LC–MS/MS to identify the interacting proteins of AMPKα1. Two unique peptides of USP5 were detected in the interacting proteins of AMPKα1, whereas no unique peptides were observed in the IgG group (*Figure* *S8a*). The Co‐IP experiments further validated the interaction between AMPKα1 and USP5 (*Figure* *6* *A*). Furthermore, we observed that the interaction between AMPKα1 and USP5 was diminished in the d‐gal group, while NaHS markedly augmented the interaction between AMPKα1 and USP5 and reduced the ubiquitination degradation of AMPKα1 (*Figure* *6* *B*). These results suggest that H_2_S inhibits the ubiquitination degradation of AMPKα1 by USP5.

To further indicate that USP5 suppresses the ubiquitination degradation of AMPKα1 and then promotes autophagy, we conducted a knockdown experiment using 150 nM USP5 siRNA (siUSP5 #1 and siUSP5 #2) in C2C12 myoblasts (*Figure* *S8b,c*). The transfection efficiency of siUSP5 #1 was more stable; thus, siUSP5 #1 was utilized for subsequent experiments. Following USP5 knockdown, there was an observable increase in the ubiquitin‐mediated degradation of AMPKα1 (*Figure* *6* *C*), accompanied by a notable reduction in AMPKα1 phosphorylation and protein abundance (*Figure* *6* *D*). Additionally, ULK1 phosphorylation and Beclin1 expression were significantly diminished, accompanied by an increase in p62 expression (*Figure* *6* *E*). Furthermore, the number of autophagosomes markedly decreased (*Figure* *6* *F*). Concurrently, the knockdown of USP5 cancelled the impact of NaHS on the aforementioned indicators (*Figure* *6* *D–F*). These findings indicate that USP5 is a deubiquitinating enzyme of AMPKα1 and that H_2_S stimulates autophagy by regulating the interaction between USP5 and AMPKα1.

### Hydrogen sulfide affects the expression and S‐sulfhydration of ubiquitin specific peptidase 5

**Figure 7:**
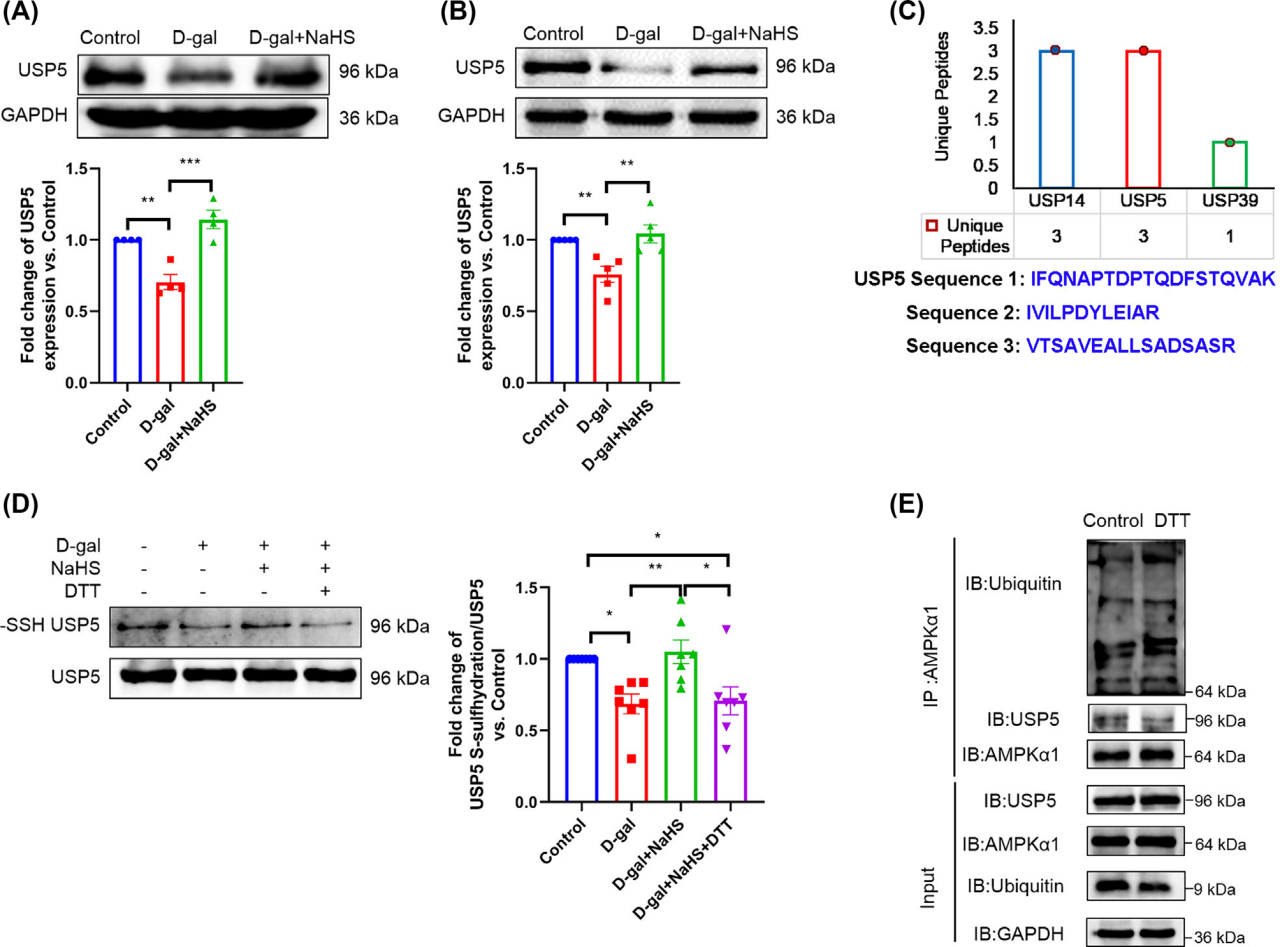
H_2_S affects the expression and S‐sulfhydration of USP5. (A, B) The expression of USP5 in gastrocnemius tissue (A, *n* = 4) and C2C12 myotubes (B, *n* = 5). (C) Members of the S‐sulfhydrated USPs family were detected by LC–MS/MS. The sequences of USP5 unique peptides detected by LC–MS/MS are shown in blue letters. (D) The S‐sulfhydration USP5 protein of C2C12 myotubes was detected with a biotin‐switch assay (*n* = 7). (E) Ubiquitination level of AMPKα1 and its interaction with USP5 in the control group and DTT group (*n* = 3). The results were expressed as the mean ± SEM. Significant differences are indicated as *P < 0.05, **P < 0.01 and ***P < 0.001. DTT, dithiothreitol.

In comparison to the d‐gal and old groups, NaHS was observed to significantly enhance USP5 expression (*Figure* *7* *A,B* and *S2e*). *Figure* *7* *C* illustrates the detection of S‐sulfhydration of USP5 in C2C12 myotubes via LC–MS/MS. Meanwhile, the S‐sulfhydration protein of USP5 decreased in the d‐gal group compared with the control group, and NaHS increased the S‐sulfhydration protein of USP5 compared with the d‐gal group. The knockdown of USP5 and DTT (an inhibitor of disulfide bonds) reversed the effect of NaHS on the S‐sulfhydration protein of USP5, and the disulfide bond was broken, resulting in a decrease in the modification of USP5 S‐sulfhydration (*Figure* *6* *D–F* and *7* *D*). Furthermore, our results indicate that DTT enhances the ubiquitination and degradation of AMPKα1, while concomitantly reducing the interaction between USP5 and AMPKα1 (*Figure* *7* *E*). Our data suggest that H_2_S promotes the expression and S‐sulfhydration of USP5, subsequently augmenting the interaction between USP5 and AMPKα1 in the ageing skeletal muscle of mice.

## Discussion

It is well known that d‐gal induces ageing by increasing oxidative stress and inducing apoptosis.^24^ The principal advantage of this approach is that it is relatively rapid and highly efficacious. For instance, researchers demonstrated that d‐gal can induce senescence in the hearts and cardiomyocytes,^11^ renal and mesangial cells,^12^ brain and gut microbiota^25^ and skeletal muscle of rats or mice.^26^ Cell senescence is characterized by three main features: increased SA‐β‐gal activity, an arrested cell cycle and increased oxidative stress.^21^ Studies have demonstrated that MuRF1 and MAFbx are transcriptionally increased in skeletal muscle under atrophy‐induced conditions, and they have been identified as excellent markers of muscle atrophy.^26^ In the present study, our findings indicate that d‐gal increased the activity of SA‐β‐gal, the level of oxidative stress and apoptosis and the expression of p21, MuRF1 and MAFbx. Conversely, it decreased the expression of Cyclin D1. Concurrently, d‐gal induced a reduction in the cross‐sectional area of muscle fibres and myolysis, accompanied by an increase in the collagen volume fraction. This indicates that the d‐gal‐induced ageing models of skeletal muscle and C2C12 myotubes were successfully established.

H_2_S, as a gas signalling molecule, plays an important role in the regulation of physiological processes in a multitude of tissues and cells throughout the body. It inhibits oxidative stress, regulates cell proliferation, apoptosis and differentiation, promotes energy metabolism and participates in signal transduction.^27^ Research conducted by our and other teams has shown that H_2_S can alleviate the effects of ageing in the heart,^11^ kidney^12^ and liver.^28^ However, it is not yet clear whether H_2_S can also inhibit the ageing of skeletal muscle. The results demonstrated a significant reduction in the production of H_2_S and the expression of CBS, CSE and 3‐MST in the d‐gal‐induced ageing gastrocnemius and C2C12 myotubes or natural ageing gastrocnemius. This demonstrates that skeletal muscle ageing may be closely related to the reduction of endogenous H_2_S. To prove this point, ageing mice and C2C12 myotubes were treated with NaHS, which resulted in an increase in H_2_S content. Concomitantly, NaHS also reversed all indications of ageing in the d‐gal group or old group. These findings indicate that skeletal muscle ageing is closely associated with a reduction in H_2_S and that H_2_S may play a role in alleviating skeletal muscle ageing.

Cells are capable of clearing harmful substances and maintaining metabolic balance through autophagy.^8^ Beclin1 plays a pivotal role in the formation of bilayer vesicle nucleation.^26^ The conversion of LC3 I to LC3 II indicates the formation of autophagosomes.^29^ P62 is specifically capable of recognizing ubiquitination proteins and the autophagosome coat protein LC3/ATG8; eventually, p62 is responsible for transporting cargos to autophagosomes and is subsequently degraded by lysosomes.^30^ The experimental data showed that d‐gal reduced the content of autophagosomes, the expression of Beclin1 and LC3 II/I and autophagic flux while enhancing the expression of p62 in ageing gastrocnemius and C2C12 myotubes. Treatment with NaHS was found to promote autophagy and the formation of autophagic flux. Concurrently, a 3‐month course of NaHS treatment was found to significantly enhance autophagy in 18‐month‐old mice. CQ was found to cancel the effect of NaHS on autophagy and ageing. These results suggest that H_2_S alleviates skeletal muscle ageing by promoting autophagy. Furthermore, we employed AICAR and LYN‐1604, agonists of the AMPKα1–ULK1 pathway, to demonstrate that H_2_S stimulates autophagy by up‐regulating the AMPKα1–ULK1 pathway.

What is the mechanism by which H_2_S regulates the AMPKα1–ULK1 pathway? In addition to phosphorylation, it has been demonstrated that AMPK can also be degraded by ubiquitination.^31^ The modification of ubiquitination is reversible, and DUB can remove the ubiquitin chain from the substrate, which is referred to as deubiquitination.^32^ USP5 is a member of the USP family and has the capacity to sever the bond between ubiquitin and substrate.^33^ Our studies indicate that USP5 is a pivotal protein that interacts with AMPKα1. Following the knockdown of USP5 in C2C12 myoblasts, the levels of AMPKα1 and p‐AMPKα1 were significantly reduced, while the ubiquitination of AMPKα1 was markedly elevated. In addition, we also found that the effects of NaHS were abolished upon USP5 knockdown. These results reveal that USP5 is a deubiquitinating enzyme of AMPKα1. H_2_S promotes the AMPKα1–ULK1 pathway by increasing the interaction between USP5 and AMPKα1.

The precise manner in which H_2_S regulates USP5 remains to be elucidated. Studies have shown that the main targets of H_2_S include the modification of proteins.^34^ S‐sulfhydration is the process by which the thiol group (–SH) of a protein‐specific cysteine residue forms a covalent bond with sulfide, polysulfide or H_2_S.^13^ S‐sulfhydration can modify specific target protein cysteine residues to regulate the biological functions of target proteins by changing their biochemical activity (activation or inhibition), stability, subcellular localization and intermolecular interactions.^13^ In this study, we detected the S‐sulfhydration of USP5 and found that treatment with NaHS significantly promoted the S‐sulfhydration of USP5, which was blocked by DTT. Meanwhile, DTT increased the ubiquitination level of AMPKα1 and diminished the interaction between USP5 and AMPKα1. We also observed that the expression of USP5 was reduced in the ageing gastrocnemius and in the C2C12 myotubes, and this was reversed by NaHS. The experimental data suggest that H_2_S enhances the interaction between USP5 and AMPKα1 by increasing the expression and S‐sulfhydration of USP5.

**Figure 8:**
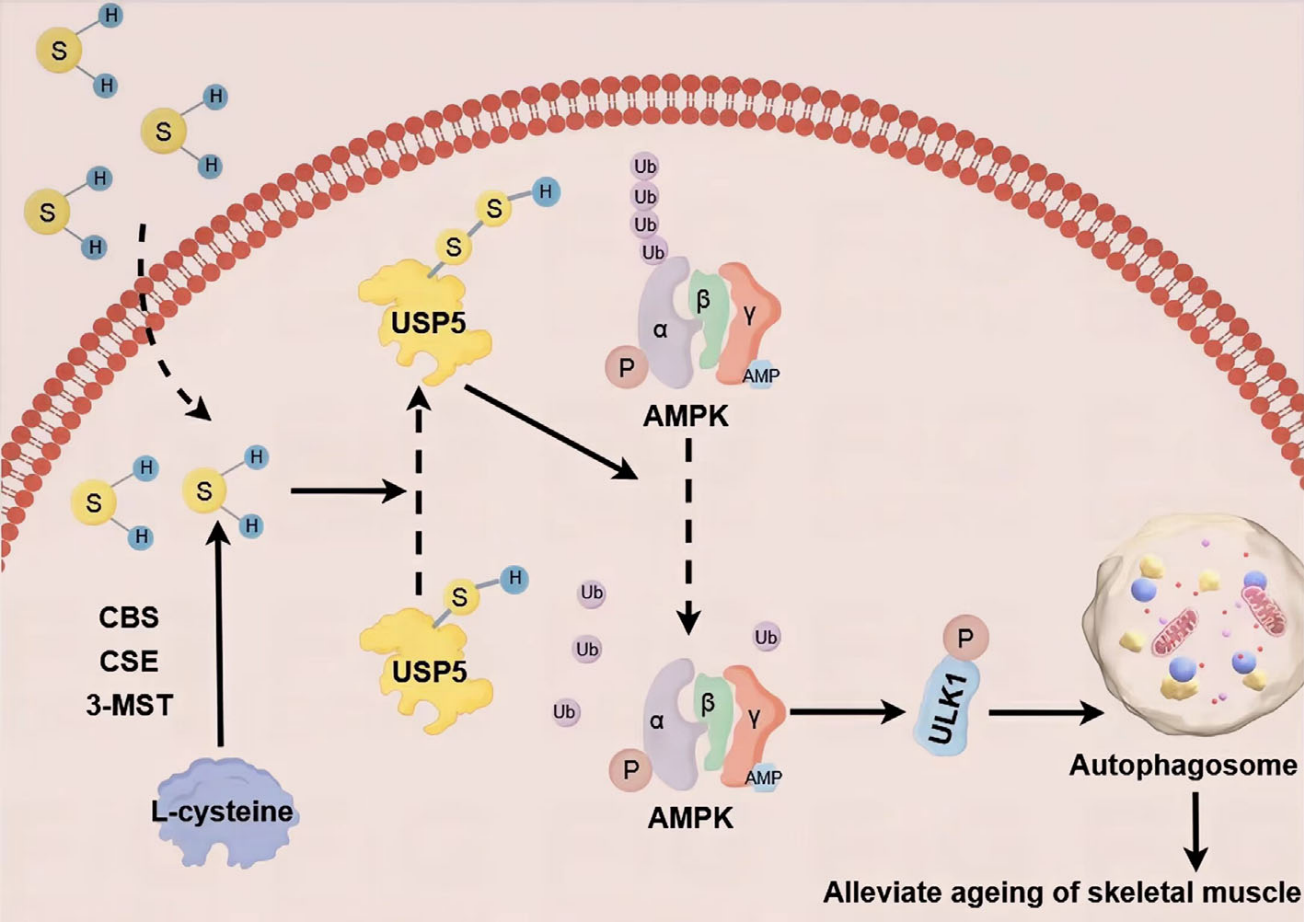
Summary of our findings in the present study. H_2_S enhances the expression and S‐sulfhydration of USP5, leading to increased deubiquitination of AMPKα1 and inhibition of its degradation. This in turn promotes the phosphorylation and activation of the AMPKα1–ULK1 pathway, ultimately up‐regulating autophagy and alleviating skeletal muscle ageing in mice.

In conclusion (*Figure* *8*), the present data demonstrate for the first time that USP5 is a deubiquitinating enzyme of AMPKα1. H_2_S increases the expression and S‐sulfhydration of USP5, subsequently promoting the deubiquitination of AMPKα1 and enhancing the phosphorylation of AMPKα1. This activates the AMPKα1–ULK1 pathway, which in turn up‐regulates autophagy. Ultimately, this alleviates the ageing of skeletal muscle. The application of H_2_S may represent a novel therapeutic strategy in the prevention and treatment of skeletal muscle ageing and atrophy.

## Funding information

This study was supported by the National Natural Science Foundation of China (No. 81770486); the Research Initiation Fund (RIF), School of Medicine, Xiamen University; the Fujian Provincial Science and Technology Guidance Program (No. 2022D033); and the Xiamen Health Care Guidance Program (No. 3502Z20224ZD1281).

## Conflict of interest statement

The authors declare no conflicts of interest associated with this manuscript.

## Supporting information


**Figure S1.** The ageing models of skeletal muscle and C2C12 myotubes in the mice.
**Figure S2.** Therapeutic effects of H_2_S on naturally ageing skeletal muscle.
**Figure S3.** H_2_S inhibits oxidative stress in ageing skeletal muscle and C2C12 myotubes.
**Figure S4.** H_2_S reduces apoptosis of ageing skeletal muscle and C2C12 myotubes.
**Figure S5.** H_2_S up‐regulates autophagy in ageing skeletal muscle and C2C12 myotubes.
**Figure S6.** H_2_S cannot alleviate ageing after CQ blocks autophagy.
**Figure S7.** Activation of AMPKα1‐ULK1 pathway can alleviate ageing of C2C12 myotubes.
**Figure S8.** 150 nM USP5 siRNA and 1/10 lipo 6000 were used to knock down USP5 in the C2C12 myoblasts.

## Data Availability

The data in the present study will be acquired from the corresponding authors according to reasonable requirements.
