# Supplementary material for: Hydrogen sulfide inhibits skeletal muscle ageing by up‐regulating autophagy through promoting deubiquitination of adenosine 5’‐monophosphate (AMP)‐activated protein kinase α1 via ubiquitin specific peptidase 5
Source: J Cachexia Sarcopenia Muscle. 2024 Aug 27;15(5):2118–33. doi: 10.1002/jcsm.13560 (PMC11446701; doi:10.1002/jcsm.13560)
Supplement: Supplementary file 1 — Figure S1. The ageing models of skeletal muscle and C2C12 myotubes in the mice. Figure S2. Therapeutic effects of H2S on naturally ageing skeletal muscle. Figure S3. H2S inhibits oxidative stress in ageing skeletal muscle and C2C12 myotubes. Figure S4. H2S reduces apoptosis of ageing skeletal muscle and C2C12 myotubes. Figure S5. H2S up‐regulates autophagy in ageing skeletal muscle and C2C12 myotubes. Figure S6. H2S cannot alleviate ageing after CQ blocks autophagy. Figure S7. Activation of AMPKα1‐ULK1 pathway can alleviate ageing of C2C12 myotubes. Figure S8. 150 nM USP5 siRNA and 1/10 lipo 6000 were used to knock down USP5 in the C2C12 myoblasts. [file JCSM-15-2118-s001.docx]

**Hydrogen sulfide inhibits skeletal muscle ageing by up-regulating autophagy through promoting deubiquitination of adenosine 5’-monophosphate (AMP)-activated protein kinase α1 via ubiquitin specific peptidase 5**

Jia-He Yang ^1, 2 #^, Jun Gao ^3 #^, Ya-Qi E ^1, 2^, Li-Jie Jiao ^1, 2^, Ren Wu ^1, 2^, Qiu-Yi Yan ^1, 2^, Zi-Yi Wei^1, 2^, Guo-Liang Yan ^1, 2^, Jin-Long Liang^4, *^, Hong-Zhu Li ^1, 2 *^

^1^ Institute of Cardiovascular Diseases, Xiamen Cardiovascular Hospital of Xiamen University, School of Medicine, Xiamen University, Xiamen, Fujian, 361006, China;

^2^ Department of Pathophysiology, School of Medicine, Xiamen University, Xiamen, Fujian, 361102, China.

^3^ Department of emergency medicine, Xiangan Hospital of Xiamen University, Xiamen, Fujian, 361101, China.

^4^ Department of General Surgery, Xiamen Fifth Hospital, Xiamen, Fujian, 361101, China

**Running title:** H_2_S alleviates skeletal muscle ageing.

^*^Correspondence to: Hong‐Zhu Li, Institute of Cardiovascular Diseases, Xiamen Cardiovascular Hospital of Xiamen University, School of Medicine, Xiamen University, Xiamen, Fujian 361006, China, and Department of Pathophysiology, School of Medicine, Xiamen University, Xiamen, Fujian 361102, China. Email: [lhz2020@xmu.edu.cn](mailto://lhz2020@xmu.edu.cn);

Jin‐Long Liang, Department of General Surgery, Xiamen Fifth Hospital, Xiamen, Fujian 361101, China. Email: [liang111@163.com](mailto://liang111@163.com)

^#^ Jia-He Yang and Jun Gao contributed equally to this study.

E-mail address: [lhz2020@xmu.edu.cn](mailto:lhz2020@xmu.edu.cn) (H. –Z. Li); [liang111@163.com](mailto:liang111@163.com) (J.-L. Liang)

Tel: +865922188672; +865927212120

**Supplementary materials**

**Supplementary methods**

*Methods S1 Reagents*

D-galactose (D-gal), Sodium hydrogen sulfide (NaHS) and 7-azido-4-methylcoumarin (C-7Az, H_2_S probe) were purchased from Sigma (MO, USA). Chloroquine (CQ, an autophagy inhibitor), Acadesine (AICAR, an AMPK agonist), LYN-1604 (a ULK1 agonist), a De-sulfhydration reagent dithiothreitol (DTT, an inhibitor of disulfide bond) and streptavidin magnetic beads were obtained from MedChemExpress (Shanghai, China). Anti-Cyclin D1 (1:1000), anti-cystathionine-β-synthetase (CBS) (1:1000), anti-3-mercaptopyruvate sulfurtransferase (3-MST) (1:1000), anti-phospho-adenosine 5’-monophosphate (AMP)-activated protein kinase α1-Thr172 (p-AMPKα1) (1:500), anti-uncoordinated-51 like kinase 1 (ULK1) (1:1000), anti-Cleaved-caspase 3 (1:1000), anti-Cleaved-caspase 9 (1:1000), anti-B-cell leukemia/lymphoma 2 (Bcl2) (1:1000), anti-muscle-specific RING finger protein 1 (MuRF1) (1:1000), anti-muscle atrophy F box protein (MAFbx) (1:1000) and protein A/G plus magnetic beads were purchased from ABclonal (Wuhan, China). Anti-AMPK α1 (1:1000), anti-sequestosome 1 (SQSTM1/p62) (1:1000), anti-Beclin1 (1:1000), anti-microtubule-associated protein 1 light chain 3 II/I (LC3 II/I) (1:1000), anti-cyclin-dependent kinase inhibitor 1A (p21) (1:1000), anti-myosin heavy chain (MHC) (1:1000), anti-cystathionine-γ-lyase (CSE) (1:1000) were obtained from Proteintech (Wuhan, China). The antibody of USP5 (1:1000) was obtained from Gentex (Calif, USA). The antibody of Phospho-ULK1-ser555 (1:1000) was purchased from ImmunoWay Biotechnology (Calif, USA). Senescence β-galactosidase (SA-β-Gal) staining kit, reactive oxygen species (ROS) assay kit, TUNEL staining kit, Hoechst 33342 staining kit, autophagy staining assay kit with monodansylcadaverine (MDC) and BCA protein assay kit were brought from Beyotime (Shanghai, China). Superoxide dismutase (SOD) assay kit, malondialdehyde (MDA) assay kit, and catalase (CAT) assay kit were obtained from Nanjing Jiancheng (Nanjing, China). RIPA lysis buffer, Phenylmethanesulfonyl fluoride (PMSF), Phosphatase inhibitor cocktail (PIC) were obtained from Meilunbio (Dalian China). Normal control siRNA, USP5 siRNA are purchased from GenePharma (Shanghai, China).

*Method S2 Western blot*

The appropriate quantity of RIPA lysis buffer (1 % PMSF, 1 % PIC) was added to the samples of gastrocnemius tissue and C2C12 myotubes. The samples were then subjected to a crushing and centrifugation process, resulting in the separation of the supernatant. The supernatant was then boiled with a 1% SDS sample buffer. The proteins were then separated on SDS-PAGE and electro-transferred to polyvinylidene fluoride (PVDF) membranes. Following a 1 h incubation period in milk, the membranes were exposed to primary antibodies at 4°C overnight. This was followed by a 1 h incubation period with secondary antibodies at room temperature. After washing with TBST buffer, the membranes were combined with the hypersensitive chemiluminescence reagent and detected by ChemiDoc XRS+ imaging system.

*Method S3 Immunofluorescence*

The frozen sections of gastrocnemius tissue were permeabilized with 0.3% Triton X-100 for 10 min. Following a 30 min wash with phosphate-buffered saline (PBS), the tissue was blocked with 5% donkey serum for 30 min. Thereafter, the sections were incubated with anti-myosin heavy chain (MHC) antibody (1:200) at 4°C overnight. On the following day, the tissue was treated with a secondary antibody conjugated to fluorescein at room temperature for 1 h. Following this, the tissue was washed with PBS and a cover glass was placed over it. The fluorescence was then observed under a fluorescence microscope.

*Method S4 Senescence-associated β-galactosidase (SA-β-gal) staining*

The senescence of C2C12 myotubes was detected using a SA-β-gal staining kit. Following a PBS wash, 1 mL of β-galactosidase staining fixing solution was added to each well for 15 min. Subsequently, 1 mL of the working solution (configure according to the instructions) was added to each well, and the C2C12 myotubes were incubated at 37°C for 12 h. The images were captured using a microscope, and the ratio of β-galactosidase-positive area to total area was measured using Image J.

*Method S5 H&E and Masson staining*

The gastrocnemius tissue was fixed in 10% neutral buffered formaldehyde and dehydrated through a serial alcohol gradient, after which it was embedded in paraffin blocks. Paraffin sections of 4 μm thickness were roasted at 60°C for 30 min, dewaxed in xylene, and hydrated through a series of decreasing ethanol concentrations. A general histopathological evaluation was conducted using H&E staining, which revealed the presence of muscle fibres within the tissue. Masson staining was employed to further delineate these fibres. The Image J software was employed to calculate the muscle cross-sectional area. A total of 20 fibres were selected for each image to calculate the average value, and six images were collected for each group.

*Method S6 Biochemical assays*

Gastrocnemius tissue and C2C12 myotubes were lysed in accordance with the instructions provided in the kit. The lysate was then centrifuged at 12,000 rpm for 10 min. The supernatant was utilized to quantify the activities of catalase (CAT), superoxide dismutase (SOD), SA-β-gal, and the content of malondialdehyde (MDA) in each sample in accordance with the provided instructions.

*Method S7 ROS content determination*

The ROS Assay Kit was used to detect the ROS levels in gastrocnemius and C2C12 myotubes. The DCFH-DA probe was diluted to 10 μmol/L with DMEM/PBS, and the configured probe was incubated with the sample at 37°C for 20 min. The probe that did not enter the cell was removed by being washed with DMEM/PBS 3 times. The fluorescence intensity was observed under a fluorescence microscope.

*Method S8 TUNEL and Hoechst 33342 staining*

The apoptosis of gastrocnemius was detected by TUNEL staining. Hoechst 33342 staining was used to detect apoptosis of C2C12 myotubes. After the slices or cells were fixed with 4 % paraformaldehyde, they were incubated with TUNEL working solution at room temperature for 20 min, or with Hoechst 33342 staining solution at 37°C for 30 min. After washing the staining solution with PBS, the fluorescence intensity was observed under a fluorescence microscope.

*Method S9 Transmission electron microscopy*

Fresh gastrocnemius tissue was treated with glutaraldehyde and 1% osmium tetroxide, dehydrated through a gradient of ethanol, and embedded in resin. The embedded tissue was sliced, and fixed with uranyl acetate and lead citrate, dried, and placed on copper plates. Autophagosomes were observed under a transmission electron microscope (TEM).

*Method S10 Monodansylcadaverine (MDC) staining*

The MDC was employed as a probe to assess the autophagy level of C2C12 myotubes. Following the administration of the various treatments, the cells were rinsed three times with assay buffer. Subsequently, 1 mL of the MDC solution (1:1000) was added to each well and incubated in an incubator for 30 min. Following the rinsing of the MDC with the assay buffer, the images were observed under a fluorescence microscope.

*Method S11 siRNA transfection*

The cells were plated to form 30-50% confluent monolayers for siRNA transfection. A solution of 15 μL siRNA was prepared by mixing it with 125 μL of DMEM (150 nM, 300 pmol siRNA) and incubated for 5 min. Subsequently, 30 μL lipo6000 was mixed with 125 μL DMEM at a ratio of 10:1, and the siRNA solution was then mixed with lipo6000 solution for 20 min. The mixture was added to a 6-well plate and incubated in an incubator for 4–6 h to facilitate siRNA transfection. Following transfection, the cells were cultured in DMEM medium containing 2% fetal bovine serum and no streptomycin-penicillin antibody, and the incubation was continued for 48 h to allow for subsequent experiments.

**Supplementary Figures:**

**
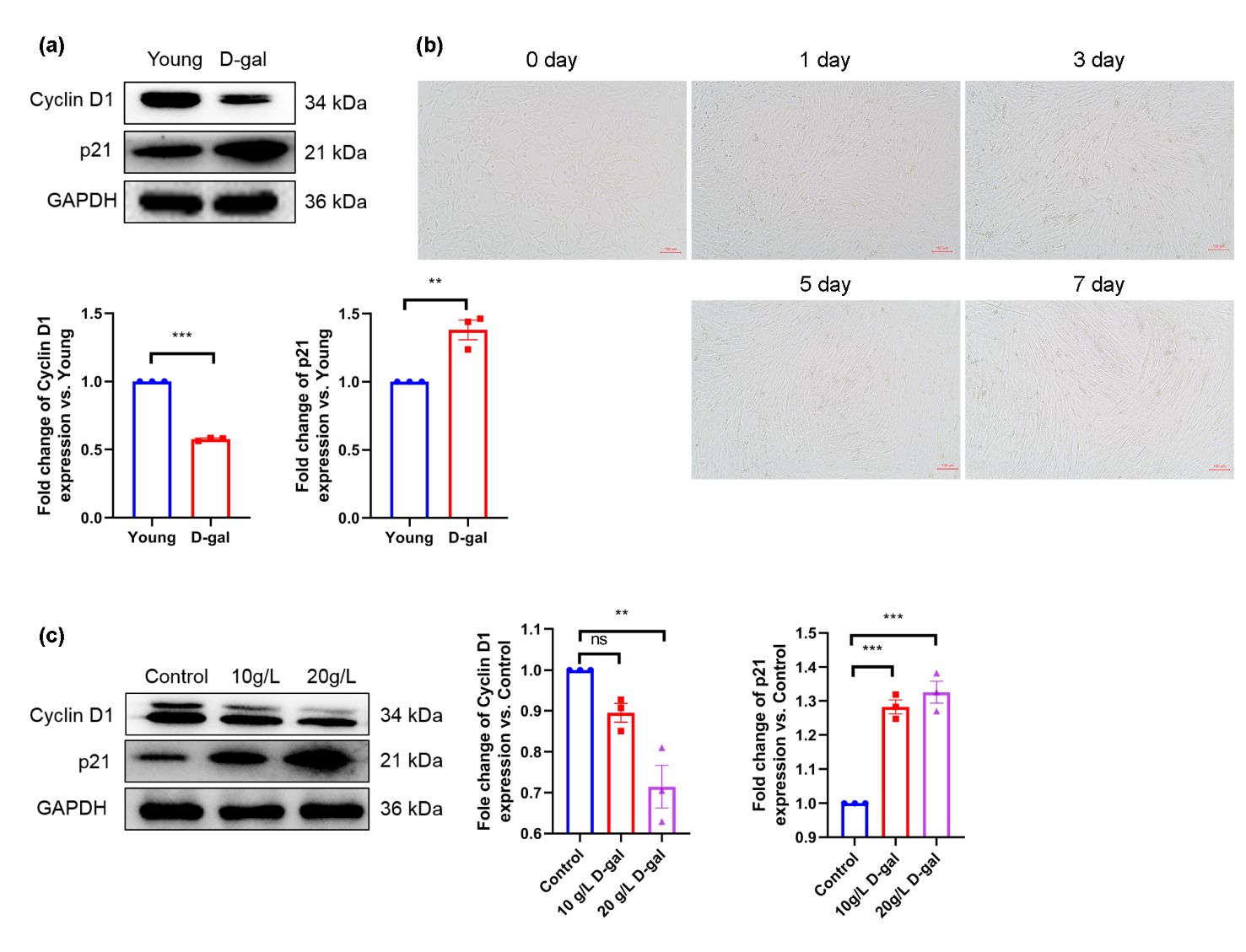
**

**Fig. S1 The ageing models of skeletal muscle and C2C12 myotubes in the mice.** **(a)** The expression of Cyclin D1 and p21 in gastrocnemius tissues (n=3). **(b)** The representative images of C2C12 myoblasts. The C2C12 myoblasts were cultured in differentiate medium for 0, 1, 3, 5, 7 days (Scale bar, 100 μm; magnification, × 100, n=6). **(c)** The expression of Cyclin D1 and p21 in C2C12 myotubes treated with different concentrations of D-gal (n=3). The results were expressed as mean ± SEM. Significant differences are indicated as ** P < 0.01, *** P < 0.001.


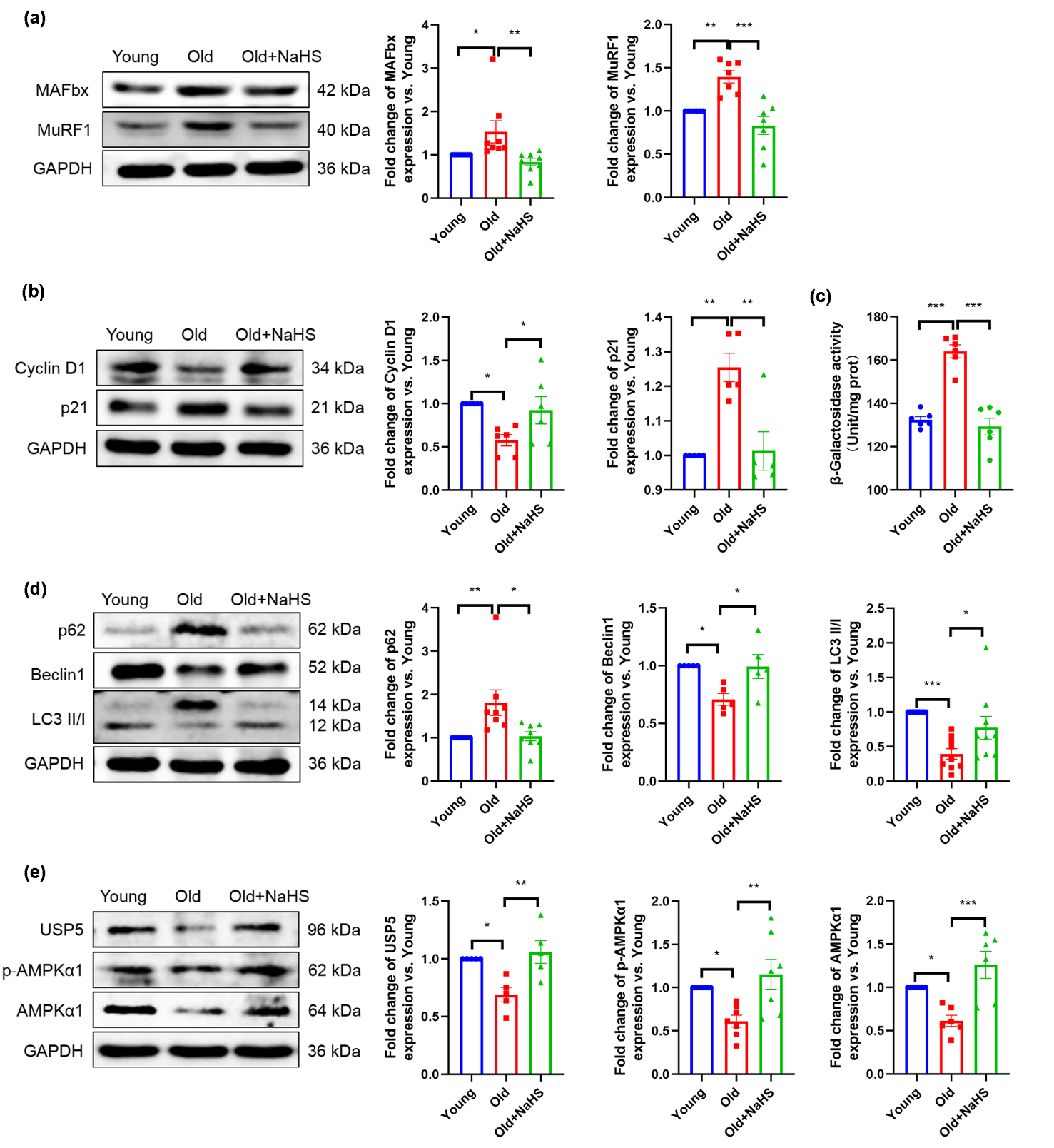


**Fig S2. Therapeutic effects of H_2_S on naturally ageing skeletal muscle. (a)** The expression of MAFbx (n=8) and MuRF1 (n=7) in gastrocnemius tissue. **(b)** The expression of Cyclin D1 (n=6) and p21 (n=5) in gastrocnemius tissue. **(c)** SA-β-gal activity of gastrocnemius tissue was measured by biochemical assay kits (n=6). **(d)** The expression of p62 (n=8), Beclin1 (n=5) and LC3II/I (n=9) in gastrocnemius tissue. **(e)** The expression of USP5 (n=5), p-AMPKα1 (n=7) and AMPKα1(n=6) in gastrocnemius tissue. The results were expressed as mean ± SEM. Significant differences are indicated as * P < 0.05, ** P < 0.01, *** P < 0.001.


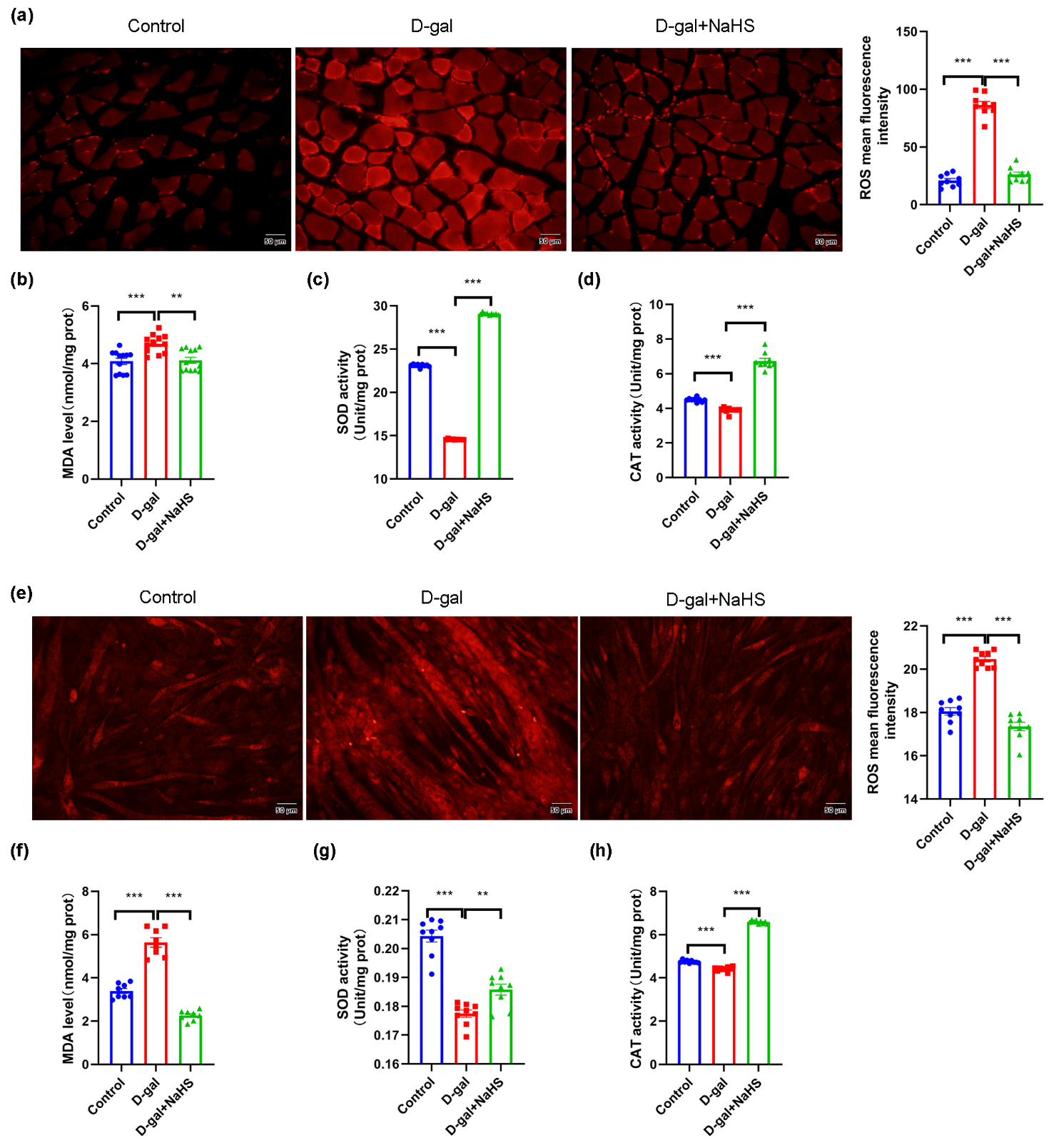


**Fig. S3 H_2_S inhibits oxidative stress in ageing skeletal muscle and C2C12 myotubes. (a, e)** Content of ROS (red) was detected by ROS staining in gastrocnemius tissue **(a, n=9)** and C2C12 myotubes **(e, n=9)** (Scale bar, 50 μm; magnification, × 200). **(b, f)** Content of MDA was measured by biochemical kits in gastrocnemius tissue **(b, n=9)** and C2C12 myotubes **(f, n=9)**. **(c, g)** Activity of SOD was tested by biochemical assay kits in gastrocnemius tissue **(c, n=9)** and C2C12 myotubes **(g, n=9)**. **(d, h)** Activity of CAT was observed by biochemical assay kits in gastrocnemius tissue **(d, n=9)** and C2C12 myotubes **(h, n=9)**. The results were expressed as mean ± SEM. Significant differences are indicated as ** P < 0.01, *** P < 0.001. CAT, catalase; MDA, malondialdehyde; ROS, reactive oxygen species; SOD, superoxide dismutase.


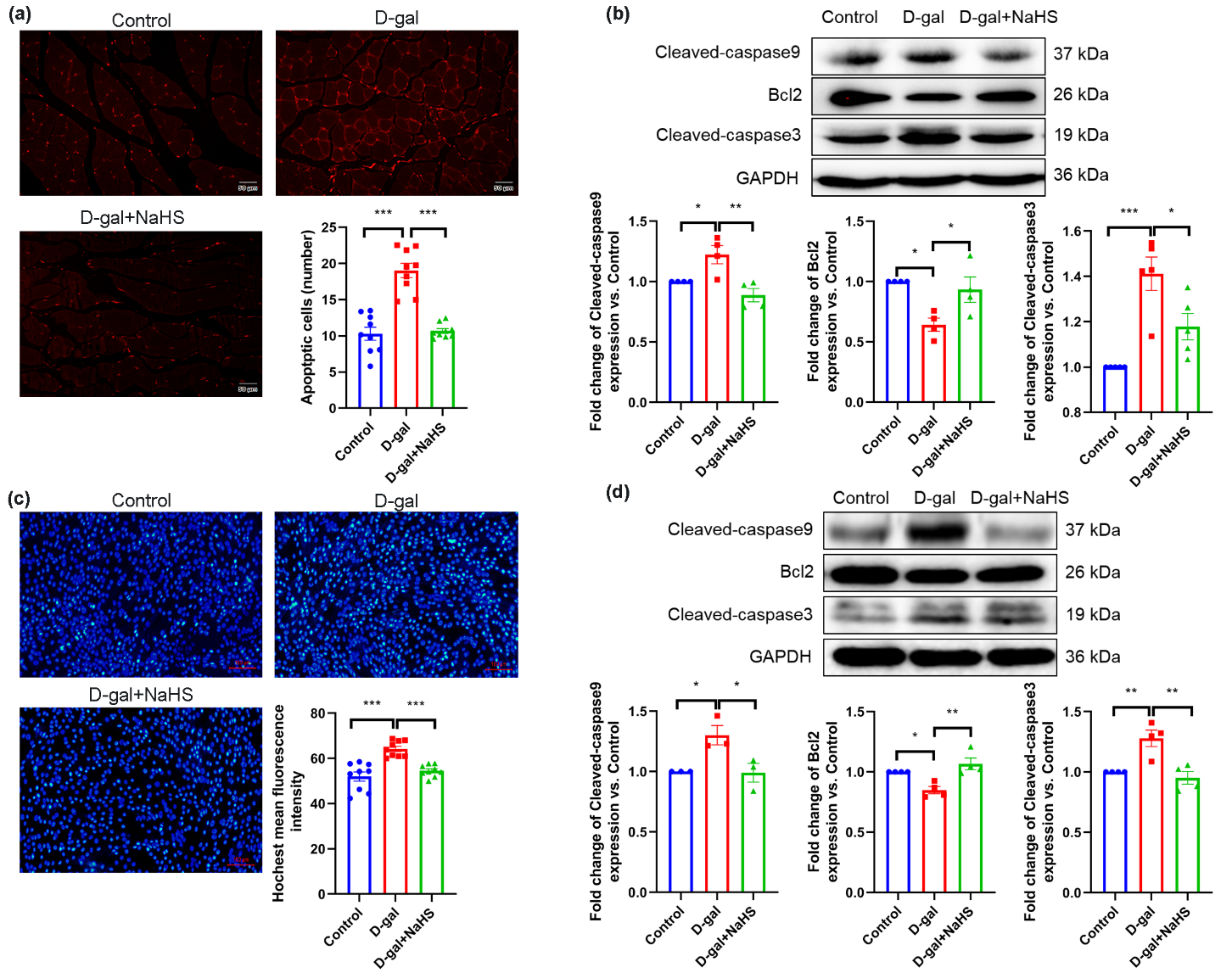
**Fig. S4 H_2_S reduces apoptosis of ageing skeletal muscle and C2C12 myotubes.** **(a)** Apoptotic cells (red) in gastrocnemius tissues were detected by TUNEL staining (Scale bar, 50 μm; magnification, × 200, n=9). **(b)** The expression of Cleaved-caspase9 (n=4), Cleaved-caspase3 (n=5) and Bcl2 (n=4) in gastrocnemius tissues. **(c)** Apoptotic cells (blue) in C2C12 myotubes were detected by Hochest staining (Scale bar, 100 μm; magnification, × 100, n=9). **(d)** The expression of Cleaved-caspase9 (n=3), Cleaved-caspase3 (n=4) and Bcl-2 (n=4) in C2C12 myotubes. The results were expressed as mean ± SEM. Significant differences are indicated as * P < 0.05, ** P < 0.01, *** P < 0.001. Bcl2, B-cell leukemia/lymphoma 2.


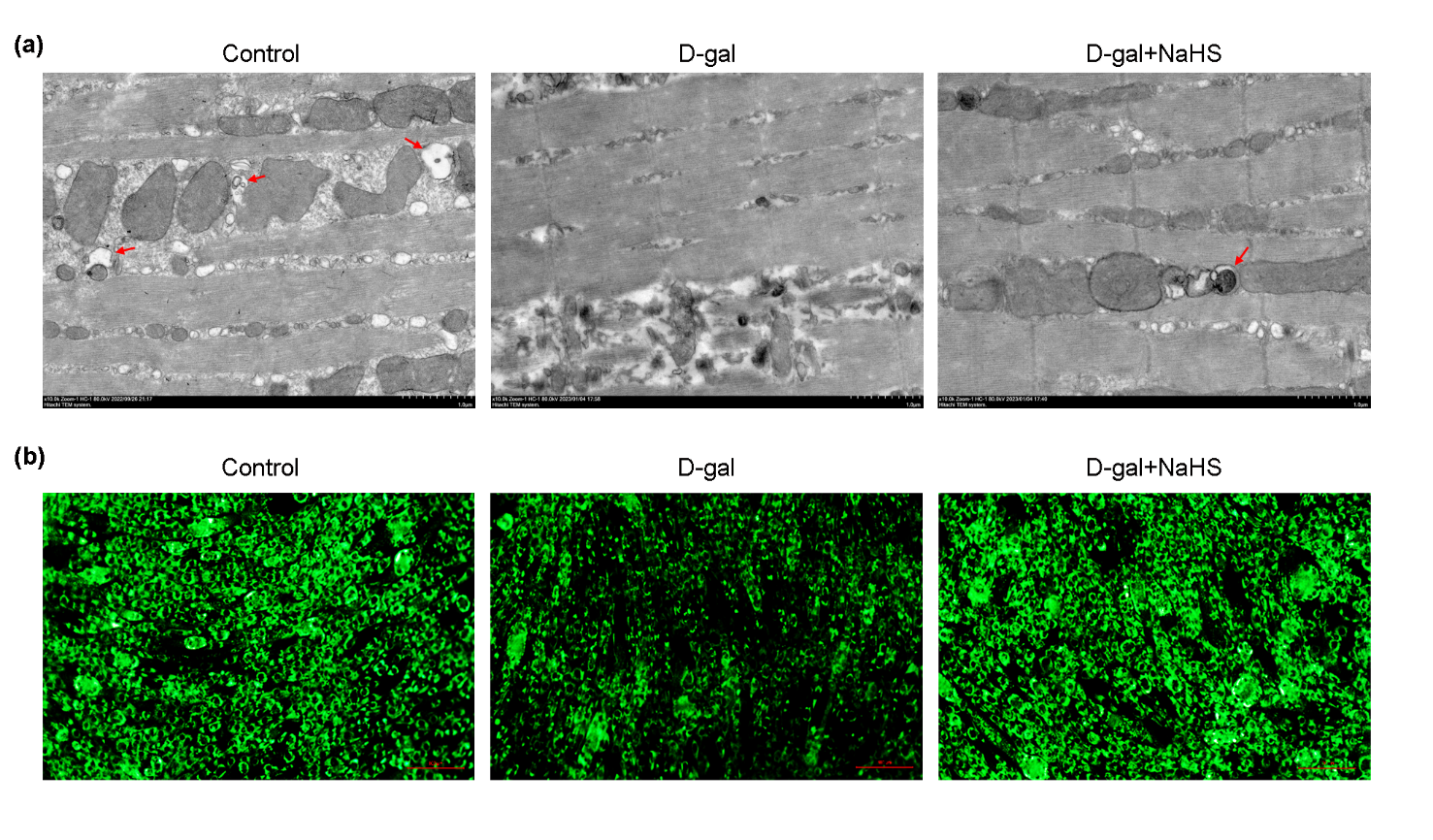


**Fig. S5 H_2_S up-regulates autophagy in ageing skeletal muscle and C2C12 myotubes.** **(a)** Autophagosomes were observed by TEM in the gastrocnemius tissue (Scale bar, 2 μm; magnification, × 20000, n=4). **(b)** Representative pictures of MDC staining in C2C12 myotubes (Scale bar, 100 μm; magnification, × 100, n=9). TEM, transmission electron microscope.


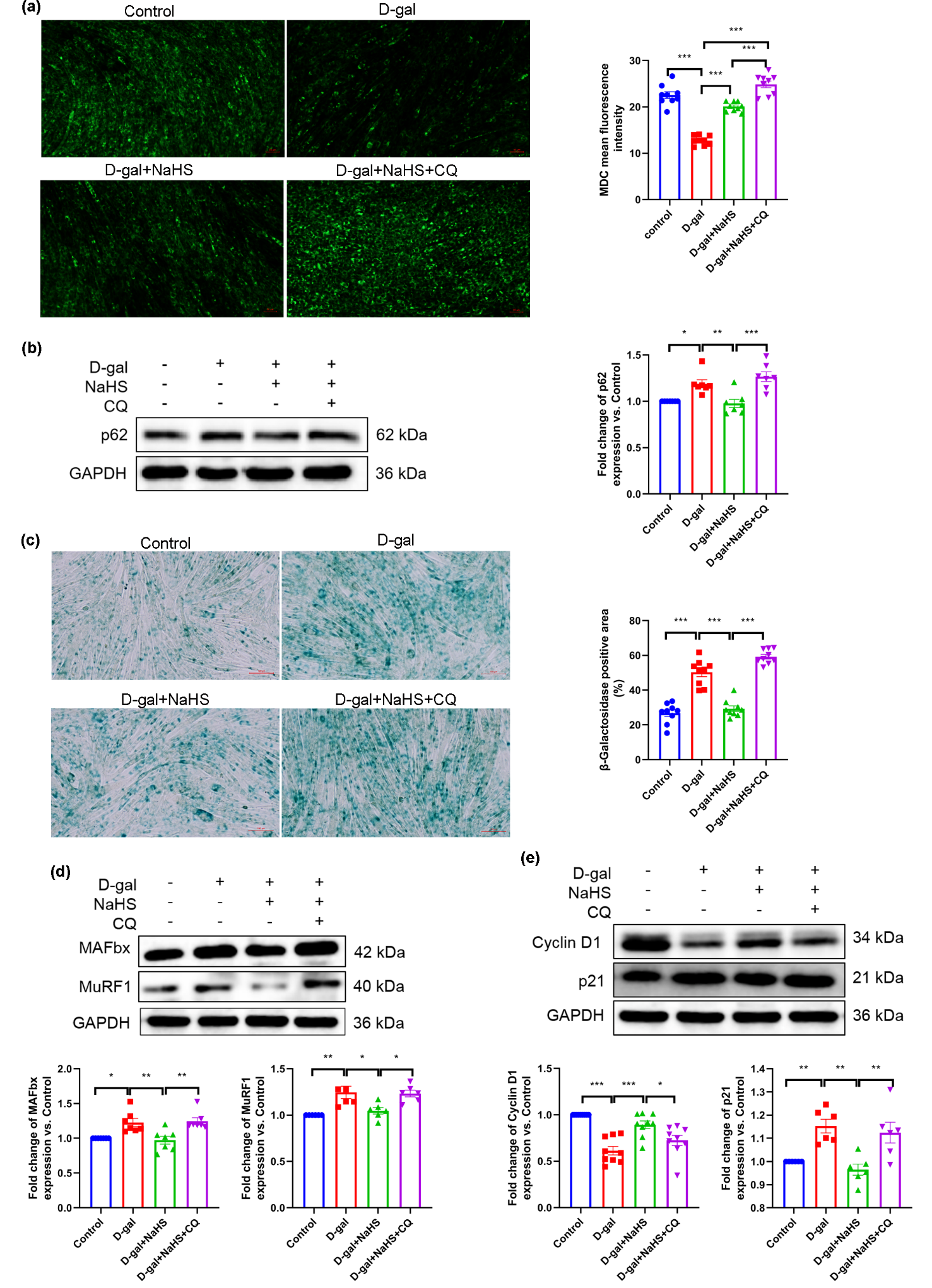


**Fig. S6 H_2_S cannot alleviate ageing after CQ blocks autophagy. (a)** Autophagosomes (green) of the C2C12 myotubes were detected by MDC staining (Scale bar, 50 μm; magnification, × 100, n=9). **(b)** The expression of p62 in C2C12 myotubes (n=7). **(c)** Representative pictures of SA-β-gal staining in the C2C12 myotubes, β-galactosidase positive areas are blue. (Scale bar, 100 μm; magnification, × 100). **(d)** The expression of MuRF1 (n=7) and MAFbx (n=6) in C2C12 myotubes. **(e)** The expression of Cyclin D1 (n=9), p21 (n=6) in C2C12 myotubes. The results were expressed as mean ± SEM. Significant differences are indicated as * P < 0.05, ** P < 0.01, *** P < 0.001.


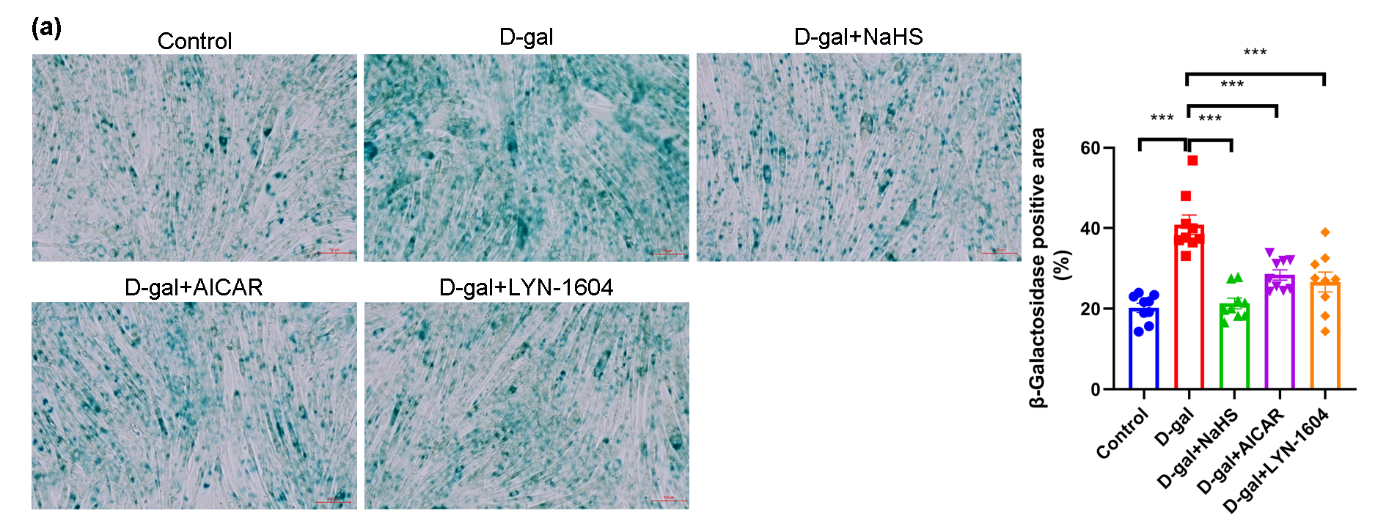


**Fig. S7 Activation of AMPKα1-ULK1 pathway can alleviate ageing of C2C12 myotubes.** Representative pictures of SA-β-gal staining in C2C12 myotubes, β-galactosidase positive cells are blue. (Scale bar, 100 μm; magnification, × 100, n=9). The results were expressed as mean ± SEM. Significant differences are indicated as *** P < 0.001.


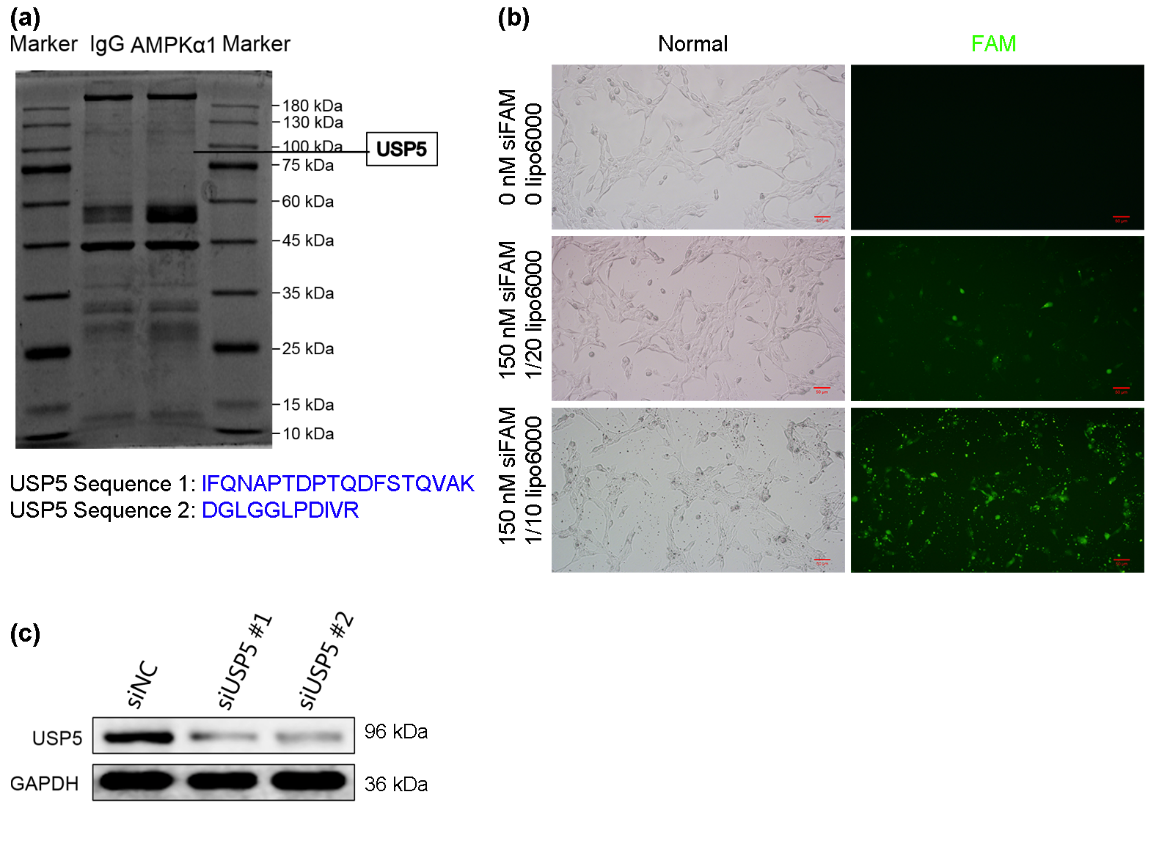


**Fig. S8 150 nM USP5 siRNA and 1/10 lipo 6000 were used to knock down USP5 in the C2C12 myoblasts. (a)** Proteins immunoprecipitated with anti-AMPKα1 and anti-IgG were detected by Coomassie blue staining and LC-MS/MS. The sequences of USP5 unique peptides that detected by LC-MS/MS are shown in blue letters. **(b)** Transfection efficiency of FAM siRNA and lipo 6000 at different concentrations was observed by fluorescence microscopy (Scale bar, 50 μm; magnification, × 200, n=3). **(c)** Transfection efficiency of USP5 siRNA #1 (siUSP5 #1) and USP5 siRNA #2 (siUSP5 #2) was detected by Western blot (n=3).
